# The relative contributions of infectious and mitotic spread to HTLV-1 persistence

**DOI:** 10.1371/journal.pcbi.1007470

**Published:** 2020-09-17

**Authors:** Daniel J. Laydon, Vikram Sunkara, Lies Boelen, Charles R. M. Bangham, Becca Asquith

**Affiliations:** 1 MRC Centre for Global Infectious Disease Analysis, Department of Infectious Disease Epidemiology, School of Public Health, Imperial College London, London, United Kingdom; 2 Section of Immunology, Wright-Fleming Institute, Imperial College School of Medicine, London, United Kingdom; 3 Department of Mathematics and Computer Science, Freie Universität, Arnimallee, Berlin, Germany; ETH Zurich, SWITZERLAND

## Abstract

Human T-lymphotropic virus type-1 (HTLV-1) persists within hosts via infectious spread (*de novo* infection) and mitotic spread (infected cell proliferation), creating a population structure of multiple clones (infected cell populations with identical genomic proviral integration sites). The relative contributions of infectious and mitotic spread to HTLV-1 persistence are unknown, and will determine the efficacy of different approaches to treatment. The prevailing view is that infectious spread is negligible in HTLV-1 persistence beyond early infection. However, in light of recent high-throughput data on the abundance of HTLV-1 clones, and recent estimates of HTLV-1 clonal diversity that are substantially higher than previously thought (typically between 10^4^ and 10^5^ HTLV-1^+^ T cell clones in the body of an asymptomatic carrier or patient with HTLV-1-associated myelopathy/tropical spastic paraparesis), ongoing infectious spread during chronic infection remains possible. We estimate the ratio of infectious to mitotic spread using a hybrid model of deterministic and stochastic processes, fitted to previously published HTLV-1 clonal diversity estimates. We investigate the robustness of our estimates using three alternative estimators. We find that, contrary to previous belief, infectious spread persists during chronic infection, even after HTLV-1 proviral load has reached its set point, and we estimate that between 100 and 200 new HTLV-1 clones are created and killed every day. We find broad agreement between all estimators. The risk of HTLV-1-associated malignancy and inflammatory disease is strongly correlated with proviral load, which in turn is correlated with the number of HTLV-1-infected clones, which are created by de novo infection. Our results therefore imply that suppression of de novo infection may reduce the risk of malignant transformation.

## Author summary

There is no effective antiretroviral treatment for infection with Human T-lymphotropic virus type-1 (HTLV-1), which causes a range of inflammatory diseases and the aggressive malignancy Adult T-cell Leukaemia/Lymphoma (ATL) in approximately 10% of infected people. Within hosts the virus spreads via infectious spread (*de novo* infection) and mitotic spread (infected cell division). The relative contributions of each mechanism are unknown, and have major implications for drug development and clinical management of infection. We estimate the ratio of infectious to mitotic spread during the infection’s chronic phase using three methods. Each method indicates infectious spread at low but persistent levels after proviral load has reached set point, contrary to the prevailing view that infectious spread features in early infection only. Risk of disease in HTLV-1 infection is known to increase with proviral load, via mutations accrued from repeated infected cell division. Our analyses suggest that ongoing infectious spread may provide an additional mechanism whereby chronic infection becomes malignant. Further, because antiretroviral drugs against Human Immunodeficiency Virus (HIV) inhibit HTLV-1 infectious spread, they may reduce the risk of HTLV-1 malignancy.

## Introduction

Human T-lymphotropic virus type-1 (HTLV-1), also known as the human T cell leukaemia virus, infects an estimated 10 million people worldwide [1]. While the majority of infected individuals remain lifelong asymptomatic carriers (ACs), in ~10% the virus causes either Adult T-cell Leukaemia/Lymphoma (ATL) [2] or a range of chronic inflammatory diseases, notably a disease of the central nervous system called HTLV-1-associated myelopathy/tropical spastic paraparesis (HAM/TSP) [3]. HTLV-1 viral burden is quantified by the proviral load (PVL), defined as the number of HTLV-1 proviruses per 100 peripheral blood mononuclear cells (PBMCs). During the chronic phase of infection, PVL remains approximately constant within each host [4, 5], but varies between hosts by over four orders of magnitude; a high PVL is associated with HAM/TSP [5, 6] and ATL [7].

**Fig 1:**
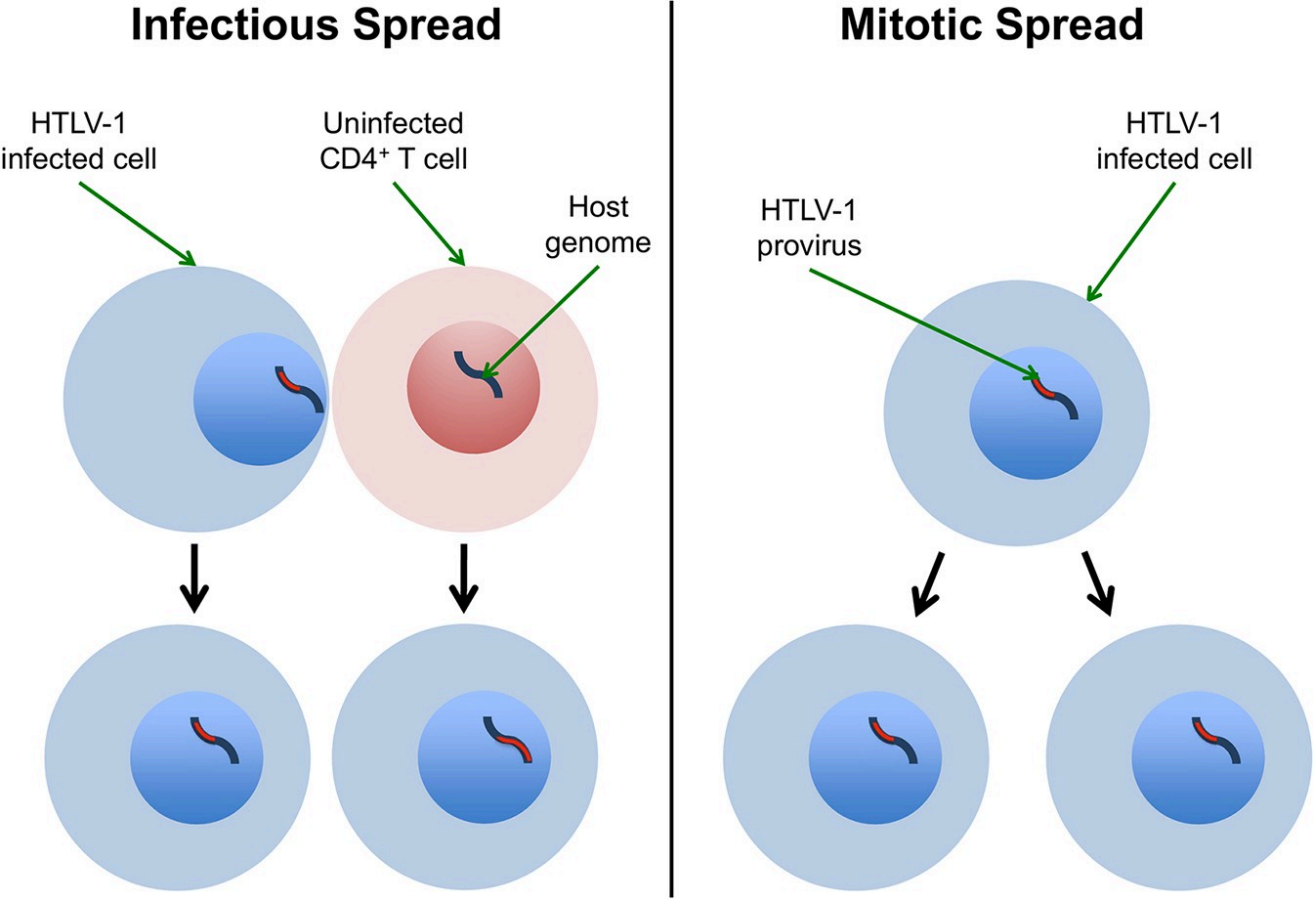
HTLV-1 infectious and mitotic spread schematic. Left column (Infectious spread): an HTLV-1-infected cell infects an uninfected CD4^+^ T cell (typically by cell-to-cell contact via the virological synapse, and potentially also via cell-free spread). The HTLV-1 provirus (red) integrates in a different genomic location in the newly infected cell, so infectious spread has resulted in two clones. Right column (Mitotic spread): An HTLV-1-infected cell divides, whereupon the provirus resides in the same genomic location in each daughter cell. The figure shows a single clone with two HTLV-1-infected cells.

HTLV-1 replicates in the host through two pathways: mitotic spread and infectious spread [8]. In mitotic spread, an infected cell divides to produce two identical “sister cells" which carry the single-copy provirus, integrated in the same genomic location as the parent cell. Infectious spread, or *de novo* infection, occurs when the virus infects a previously uninfected cell, and in this case the virus integrates in a new site in the target cell genome (Fig 1). The combination of infectious and mitotic spread results in a large number of distinct clones of infected T-cells, where each clone is defined as a population of infected cells with a shared proviral integration site [9–11].

The relative contributions of infectious spread and mitotic spread to the proviral load are unknown. This ratio is important, because it will directly determine the efficacy of different approaches to treatment. Although no effective antiretroviral drugs have yet been developed for HTLV-1 infection, antiretroviral therapy (ART) efficiently reduces infectious spread in HIV-1 infection by inhibiting reverse transcription, viral maturation and proviral integration. ART may therefore be effective in HTLV-1 infection if infectious spread contributes to HTLV-1 pathogenesis. Alternatively, drugs that inhibit T cell proliferation, such as cyclosporin, would be expected to be more useful if mitotic spread [8] is the dominant mode of viral spread.

The number of clones of HTLV-1-infected T cells depends on the extent of infectious spread. In this paper, we refer to this number as the HTLV-1 clonal “diversity" (this term should not be confused with measures such as Shannon entropy or beta diversity). The diversity in one host is unknown, and estimating this number from blood samples is nontrivial. Diversity estimation is challenging given the nature of the HTLV-1 clone frequency distribution, where the majority of infected cells are contained in relatively few clones, and the majority of clones contain relatively few cells.

The prevailing view is that mitotic spread accounts for the long-term persistence of HTLV-1 *in vivo* [11–14], and that infectious spread is negligible after initial infection [12, 13]. This belief was supported by three main observations. First, it was thought that there were relatively few (~100) HTLV-1 clones in one host [9, 11, 13, 15–19]. Second, HTLV-1 varies little in sequence both within and between hosts [20]. Since the host DNA polymerase used in cell proliferation (mitotic spread) is far less error-prone than the viral reverse transcriptase used in infectious spread, a lack of sequence variation implies that infectious spread is rare. Third, many HTLV-1^+^ clones have been observed at multiple time points separated by several years [9, 17], and a long-lived clone is very unlikely to be maintained by repeated proviral integration at the same integration site through infectious spread, especially since there are no hotspots of HTLV-1 integration [9].

However, these three observations do not necessarily imply that infectious spread is negligible in HTLV-1 pathogenesis [14], particularly when we consider the total number of clones in the host and the very small proportion of clones that can be sampled. First, the number of clones that have been both estimated and directly observed has increased over time [9, 11, 13, 15, 17, 19], and current estimates give approximately 10^4^–10^5^ clones in the circulation of ACs and patients with HAM/TSP [10, 21, 22]. Because smaller and smaller clones can be detected as method sensitivity increases, it is no longer a given that all clones are large and thus that infectious spread is non-existent during chronic infection. Second, apparent sequence uniformity may result from repeated detection of sister cells from a small number of expanded clones. That is, because of the limitations of sampling, there is a strong bias to detection of the large clones which have expanded through mitosis. Third, the repeated observation of specific clones over many years does not rule out persistent infectious spread. The observation of a temporary but dramatic PVL reduction in a patient with HAM/TSP following treatment with the reverse transcriptase inhibitor lamivudine [23] implies that infectious spread could remain important in HTLV-1 persistence, at least in some cases. Finally, the virus must retain the capacity for infectious spread, for the simple reason that it could not spread between hosts otherwise. We therefore believe it is right to question the assumption that all clones are formed during early infection.

Mitotic spread must still be predominant in proviral load maintenance, however, because the 10^4^–10^5^ clones (created by infectious spread) present in one host consist of approximately 10^11^ infected cells (created by mitotic spread). However, this consideration ignores the possibility that clones may be continuously created by infectious spread and killed by the immune response and natural death.

The aim of this study was to quantify the rate of infectious spread, and thus the ratio of infectious spread to mitotic spread during chronic infection. We first estimated HTLV-1 clonal diversity from sequence data in 11 subjects using our previously developed method [10]. We next fitted a deterministic and stochastic hybrid model of within-host HTLV-1 persistence to clonal diversity estimates. To ensure the robustness of our estimates, we used three alternative estimators of the infectious spread rate, the first of which approximates the upper bound of the infectious spread rate using a simplified model of HTLV-1 clones. The final two estimators are adapted from a method originally developed to model naïve T cell dynamics. We find broad agreement between estimates from all methods. We conclude that, during chronic infection, a given HTLV-1-infected cell in the peripheral blood is substantially more likely to be derived by mitosis of an existing clone than by de novo infection, although infectious spread continues throughout chronic infection, with an average of 175 new clones created every day.

## Methods

### Data sets

We apply all three methods described below to previously obtained high-throughput data on HTLV-1 clonality [9]. Each HTLV-1 dataset quantifies the abundance of HTLV-1-infected T cell clones in ex vivo PBMCs, without selection or culture. We studied 11 subjects, where each subject had three blood samples taken per time point, at three time points separated by an average of 4 years, giving a total of 99 datasets. All subjects either had HAM/TSP, HTLV-1-associated uveitis or were asymptomatic carriers of HTLV-1.

### HTLV-1 clonal diversity estimates

**Fig 2:**
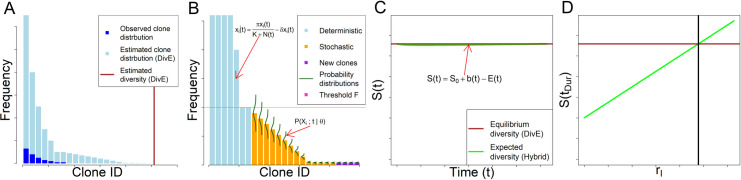
Schematic of full simulation hybrid model. **A**: Observed and estimated clone frequency distributions. From an observed sample of clones, the clone frequency distribution of the body in one host is estimated using DivE. **B**: Propagation of hybrid model: Estimated clone frequency distribution partitioned into deterministic and stochastic systems. Clones of frequency less than and greater than threshold *F* are respectively modelled stochastically and deterministically. *F* is chosen with respect to probability of clone extinction (S2 Text). The deterministic system is modelled using ordinary differential equations (Eq (1)). The stochastic system consists of multiple birth-death processes (one for each stochastically modelled clone) each with an absorbing state at zero (Fig 3A and 3B). The evolution of the clone probability distribution over time is governed by the chemical master equation (Eq (10), Fig 3C and 3D). New clones are created through infectious spread, i.e. the per-capita rate *r*_*I*_ multiplied by the expected number of infected cells, in both deterministic and stochastic compartments (Eq (11)). Deterministic and stochastic systems are propagated concurrently with Strang splitting (S2 Text). **C**: Hybrid model diversity. The estimated number of clones *S(t)* (Eq (13)) at time *t*, given parameters *θ = {π*, *δ*, *K*, *r*_*I*_*}* is given by the number of clones created (Eq (11)), minus the number of clones that are expected to have died between 0 and *t* (Eq (12)), plus the number of clones *S*_*0*_ at *t = 0*. The number of clones is assumed to be at equilibrium in the chronic phase of infection. **D**: Model fitting schematic: Expected diversity at *S(t*_*Dur*_*)* increases with per-capita infectious spread rate *r*_*I*_. Model fitted using non-linear least squares to DivE estimated diversity in the body, where the objective function is the square of the discrepancy between this value and the value of *S(t*_*Dur*_*)* at equilibrium.

To estimate the rate of infectious spread we first estimated HTLV-1 clonal diversity. We use our recently developed estimator, “DivE" [10, 24, 25], which uses experimental observations of clonal diversity in a sample to estimate both the number of clones and their frequency distribution in the body of the host (Fig 2A). DivE fits multiple mathematical models to individual-based rarefaction curves; such curves plot the expected number of clones against the number of infected cells sampled. Numerical criteria score models on their ability to accurately estimate the observed rarefaction curve when fitted to nested subsamples of the data. The best-performing models are extrapolated to estimate the total number of clones in the body, based on the proviral load in each respective subject. See [10, 25] for further details and implementation.

S2 Table gives the notation used in the three modelling approaches that follow.

### Modelling approach 1: Full simulation hybrid model

Within a given host, HTLV-1^+^ T cell clones vary in abundance by several orders of magnitude [9, 10]. Broadly, abundant clones can be modelled deterministically but small clones must be modelled stochastically. In the following sections, we describe a model of HTLV-1 dynamics at quasi-equilibrium that is a hybrid of deterministic and stochastic parts (Fig 2).

### Deterministic model

We consider a system with *S(t)* clones, where a given clone *i* has frequency *x*_*i*_*(t)* at time *t*. We have the following ordinary differential equations (ODEs) for each clone:

$$\frac{dx_{i}}{dt}=\frac{\pi x_{i}}{K+N(t)}-\delta x_{i}$$ (1)

where $N(t)=\sum_{j=1}^{S(t)}x_{j}(t)$ is the total number of infected cells summed over all clones at time *t*; $\frac{\pi }{K+N(t)}$ is the proliferation rate of infected cells (i.e. the rate of mitotic spread) which is half maximal when *N(t) = K* (S1 Text) and *δ* is the death rate of infected cells (Fig 2B). New clones appear at a rate *r*_*I*_ × *N(t)*, where *r*_*I*_ is the per-capita rate of infectious spread. Note that we make the simplifying approximation of a single proliferation rate, $\frac{\pi }{K+N(t)}$, for all clones.

The dynamics of small clones, where random effects are important, will not be adequately described by a deterministic model. Since small clones contain most information about infectious spread, it is important to model these clones accurately, and so we use a discrete stochastic model, in which we consider multiple potential states of each clone and their corresponding probabilities over time.

### Stochastic model

Using a stochastic framework, the number of clones *S(t)* and their frequencies at time *t* are considered as random variables, and we describe within-host HTLV-1 dynamics by a set of reactions and their corresponding propensities (S2 Text). Infected cells can proliferate, die, or infect uninfected cells (Fig 1). Thus the total number of possible reactions C∈ℕ at time *t* is *C = 3S(t)*. Following the formulation given in [26, 27], let *X*(*t*) = (*X*_*i*_(*t*)_*i*∈*S*(*t*)_)^*T*^ be the state vector at time *t* of all clones. *X(t)* is a random variable in ${\mathbb{N}}^{S_{\max}}$ that consists of the random variables $X_{i}(t)\in {\mathbb{N}}_{0}=\mathbb{N}\cup \{0\}$ of the frequencies *x*_*i*_*(t)* of clones *i =* 1,…, *S*_*max*_, where *S*_*max*_ is chosen to always be larger than *S(t)* for all *t*. The state vector *X(t)* evolves through a Markov jump process that depends only on the current state $y\in {\mathbb{N}}_{0}^{S_{\max}}$, and its evolution is given by

$$X(t)=y_{0}+\sum_{c=1}^{C}P_{c}\left(\int_{0}^{t}\alpha_{c}(X(s))ds\right)\nu_{c}$$ (2)

where *ν*_*c*_ and *α*_*c*_ respectively denote the stoichiometric vector and propensity function of reaction *c* [26, 27]. Eq (2) states that the population *X(t)* at time *t* is equal to the initial population *y*_*0*_ plus the sum of the changes induced by all reactions. See S2 Text for further details.

There exists a probability distribution associated with the random variable $X(t)\in {\mathbb{N}}_{0}^{S_{\max}}$ in (2), given by $\mathbb{P}(X;t)=\mathbb{P}(X(t)=y|X(0)=y_{0})$, where $y,y_{0}\in {\mathbb{N}}_{0}^{S_{\max}}$. ℙ(X;t) is a column vector where each entry is a probability associated with a potential state of the random variable at time *t*. It can be shown [27–30] that ℙ(X;t) is a solution of the Chemical Master Equation (CME)

$$\frac{\partial \mathbb{P}(X=y;t)}{\partial t}=\sum_{c=1}^{C}\left(\alpha_{c}(y-\nu_{c})\mathbb{P}(X=y-\nu_{c};t)-\alpha_{c}(y)\mathbb{P}(X=y;t)\right)$$ (3)

which describes the rate of change in the probability distribution associated with *X(t)*. The first term is the sum over all reactions of the probability of arriving at state *X(t) = y* from state *X(t) = y—ν*_*c*_ via reaction *c*, and the second term is the sum over all reactions of the probability of leaving state *X(t) = y* via reaction *c*.

**Fig 3:**
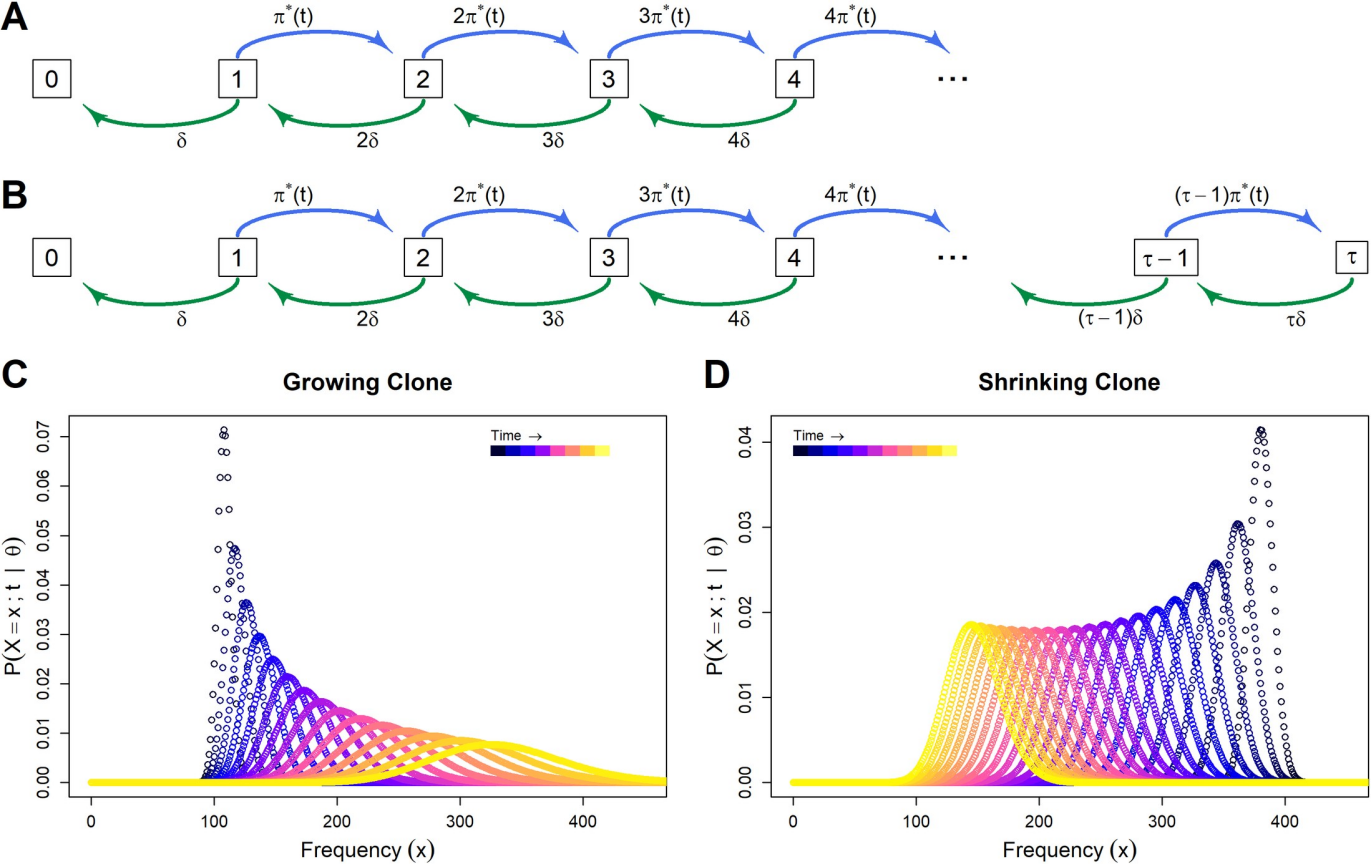
Stochastic clone dynamics. **A** and **B** respectively show the clone state space with and without an upper limit *τ*. Each box denotes the potential state of a given clone, i.e. the number of cells in that clone, with the corresponding propensity of each reaction at each state. π*(t) and *δ* denote the per-capita rates of infected cell proliferation and death respectively. Note there is no source inflow from frequency *0* to frequency *1*. **C** and **D** show clone frequency probability distributions over time. Each curve shows the distribution $\mathbb{P}(X_{i};t)=\mathbb{P}(X_{i}(t)=x_{i}|X_{i}(0)=x_{i,0})$ of the probability that the given clone *i* contains *x*_*i*_ cells at time t. At successive time points the curve broadens and either shifts to the right as the expected frequency of the clone increases **(C)**, or shifts to the left as the expected frequency of the clone decreases (**D)**.

For a single clone $X_{i}$, the following reactions respectively describe mitotic spread, cell death and infectious spread:

$$\rho_{i,1}:X_{i}\overset{\pi^{*}(t)}{\to }2X_{i}$$ (4)

$$\rho_{i,2}:X_{i}\overset{\delta }{\to }*$$ (5)

$$\rho_{i,3}:X_{i}\overset{r_{I}}{\to }X_{i}+X_{S(t)+1}$$ (6)

where

$$\pi^{*}(t)=\frac{\pi }{K+N(t)}$$ (7)

is the aggregate density-dependent proliferation rate (dependent on the carrying capacity, and the numbers of infected and uninfected cells). The first two reactions of each clone describe a birth-death process, and the lack of inflow from source (i.e. the lack of a reaction $\rho_{}:*\to X_{i}$) defines an absorbing state (Fig 3A and 3B). Note that Eq (6) makes the approximation that all instances of infectious spread lead to a new clone. Any errors from this assumption will be small, and this is considered in greater detail in the Discussion.

The reactions (4), (5) and (6) are monomolecular (in terms of the chemical master equation), because they carry the simplifying assumption that cell death due to the host immune response, and the proviral load, are each constant in the equilibrium within each host. HTLV-1 proviral load remains stable over many years [4, 5]: that is, the numbers of infected and uninfected cells stays approximately constant during the chronic phase of infection.

### Simplifying approximations of stochastic model

The probability distribution ℙ(X;t) describes the states and associated probabilities of the entire system, and we define the probability distribution of a particular clone *i*, $\mathbb{P}(X_{i};t)$ associated with the random variable *X*_*i*_*(t)* similarly: $\mathbb{P}(X_{i};t)=\mathbb{P}(X_{i}(t)=x_{i}|X_{i}(0)=x_{i,0})$, where $x_{i},x_{i,0}\in {\mathbb{N}}_{0}$. The extinction probability of clone *i* at time *t*, $\mathbb{P}(X_{i}=0;t)$, is used below to calculate the expected number of clones at time *t* (Fig 2C), which in turn enables the model to be fitted to clonal diversity estimates (Fig 2D).

If clones interact and are modelled with a single master equation associated with ℙ(X;t), the complexity and runtime of the model increase exponentially with the number of clones. However, because we model the system when proviral load is in equilibrium and can therefore use monomolecular reactions, density-dependent proliferation rates remain approximately constant, and so we can model each clone in isolation with multiple master equations associated with multiple clone-specific distributions $\mathbb{P}(X_{i};t)$ (*i =* 1, …, *S(t)*) (Fig 2B). Therefore, the model complexity and runtime increase only linearly with the number of clones.

If we impose a maximum frequency for a particular clone *i* (S2 Text, Fig 3B), we can summarise Eq (3) using multiple, simpler differential equations below

$$\frac{d\mathbb{P}(X_{i};t)}{dt}=A\mathbb{P}(X_{i};t)\;\mathrm{for}\;i=1,\ldots ,S_{max}$$ (8)

where *A* is the transition matrix or “matrix of connections" (S2 Text) [27, 31, 32]. Further, because the proliferation rate is constant at equilibrium, rates are independent of time, and so Eq (8) has solution

$$\mathbb{P}(X_{i};t)=e^{At}{\mathbb{P}}_{0,i}$$ (9)

where ${\mathbb{P}}_{0,i}=\mathbb{P}(X_{i};t=0)$ is the initial probability distribution and *e*^*At*^ is the matrix exponential [33]. For equally spaced time steps ${\left(t_{n}\right)}_{n=0}^{N}$ of length *h*, $\mathbb{P}(X_{i};t)$ can be calculated recursively

$$\mathbb{P}(X_{i};t_{n})=e^{Ah}\mathbb{P}(X_{i};t_{n-1}).$$ (10)

Example solutions of Eq (9) are shown in Fig 3C and 3D.

### Expected number of clones

We model the expected number of clones *S(t)* at time *t* by adding the total number of clone “births” *b(t)* over time (that is, the number of infectious spread events), and subtracting the total number of clone extinctions *E(t)* over time. *b(t)* is given by

$$b(t)=\int_{0}^{t}r_{I}\left[\sum_{j=1}^{b(u)}x_{j}(u)\right]du,$$ (11)

where *r*_*I*_ is the per-capita rate of infectious spread, *x*_*j*_*(t)* is the expected frequency of the *j*^th^ clone to be born since *t* = 0 (i.e. $x_{j}(t)=E\left[X_{j}(t)\right]$), and *b(*0*)* = 0. *E(t)* is then given by

$$E(t)=\sum_{j=1}^{S_{0}+b(t)}\mathbb{P}(X_{j}=0;t)$$ (12)

Note that *b(t)* and *E(t)* are increasing functions since *r*_*I*_, *x*_*j*_*(t)* ≥ 0, and because a clone frequency of zero is an absorption state for the random variable *X*_*j*_*(t)*. Taking (11) and (12) together we calculate the number of clones *S(t)* as

$$S(t)=S_{0}+b(t)-E(t)$$ (13)

where *S*_*0*_ is the number of clones at time zero (Fig 2C).

Eq (11) does not have a density-dependent *r*_*I*_, and so for large values of the number of new clones and cells would tend to infinity over time. Practically, however, *r*_*I*_ is sufficiently small that it was unnecessary to add density-dependent *de novo* infection to the model.

### Hybrid model fitting and uncertainty

It is estimated that there are approximately 10^11^ HTLV-1 infected cells in one host [10], and so it is not computationally feasible to model all clones using our stochastic formulation. Clones above a certain frequency [*F* = 460 cells; S2 Text] are assumed to be adequately described by the expected value from the deterministic ODEs in Eq (1) (Fig 2B, 2C and 2D). We thus partition our system of HTLV-1 within-host dynamics into a deterministic system of ODEs, and a stochastic system of master equations (Fig 2B). We propagate these systems alternatively and concurrently using “Strang splitting" (S2 Text) [34]. The deterministic system described in Eq (1) has an ordinary differential equation for each clone. Since *S(t)* can exceed 10^5^, we group clones into categories based on the order of magnitude of their abundance.

We model the dynamics of clones in the body, and not only the blood, because this allows us to model clone extinction. If zero cells of a particular clone are observed or estimated in the blood, this does not necessarily imply that the clone is extinct, because cells in that clone could remain in the solid lymphoid tissue, which contains 98% of lymphocytes. We model clones in the body as a whole to avoid this difficulty, which necessitates the assumption that the clonal population structure in the blood is representative of the HTLV-1 clonal structure in the whole body.

We fitted the infectious spread rate *r*_*I*_ as a free parameter, with all other parameters (infected cell proliferation rate, death rate and density dependency) fixed using previous results from the literature and based on each subject’s proviral load [35] (S1 Text). For each subject sample and parameter update of *r*_*I*_, the model was run to reach an approximate equilibrium (Fig 2C). The model was fitted to the estimated clonal diversity of that subject sample, i.e. to determine the value of *r*_*I*_ required to keep the clonal diversity at the observed equilibrium value (Fig 2D).

The uncertainty in the estimate of *r*_*I*_, the rate of infectious spread, derives from three sources: error in model choice (both structure and numerical value of fixed parameters), error in clonal diversity estimation, and sampling variation. Classical methods of quantifying fitted parameter uncertainty only reflect the last source of error (i.e. they assume that the model and the data are correct). We address the first difficulty by using three alternative models with different structures and parameters, and through a sensitivity analysis on the infected cell proliferation and death rates (S3 Text). We address the error in diversity estimation by using alternative clonal diversity inputs from the Chao1 estimator [36], a non-parametric diversity (or species richness) estimator that has been widely used in many fields [37–40]. And we address the issue of sampling variation by investigating the range of estimates provided by the nine hybrid model fits per subject (i.e. one for each of the subject's blood samples); the mean of these estimates is taken as our point estimate.

The hybrid model was coded in R (version 3.5.0) [41], using the packages “data.table" [42] and “Matrix" [43]. Matrix exponentials were computed using the Padé approximation [44]. The hybrid was fitted using one-dimensional optimisation as described in [45]. The model code is available at https://github.com/dlaydon/HTLV_1_InfMit_Hybrid.

### Modelling approach 2: Upper bound approximation

We considered a simplified model of HTLV-1 persistence that does not describe individual clone dynamics. If *S(t)* and *N(t)* are the number of clones and number of infected cells respectively at time *t*, and *r*_*I*_, is the per-capita rate of infectious spread, we have the following differential equation

$$S^{\prime }(t)=r_{I}N(t)-\delta_{S}(t)S(t)$$ (14)

where *δ*_*S*_*(t)* is the *clone* death rate at time *t*. The first term of Eq (14) models the birth of new clones by infectious spread, and the second term models the death of existing clones.

If *δ* is the (constant) death rate of infected *cells*, then we have *δ*_*S*_*(t)* ≤ *δ*, because the number of clones that die cannot exceed the number of cells that die (equality would occur if all clones were singletons i.e. clones that contain only one infected cell). The clone death rate depends on the population structure of infected cells and will vary over time as this population structure changes. For example, a higher proportion of singletons will increase *δ*_*S*_*(t)*.

We assume that, in the chronic stage of infection when HTLV-1 proviral load is at equilibrium, the number of clones is also at equilibrium and so we have *N(t) = N*, *S’(t) = 0*, and *S(t) = S*. Letting $\hat{\delta_{S}}$ be the average rate of clone death, we can approximate Eq (14) as

$$S^{\prime }(t)=0=r_{I}N-\hat{\delta_{S}}S$$ (15)

$$\Rightarrow r_{I}=\frac{\hat{\delta_{S}}S}{N}\leq \frac{\delta S}{N}$$ (16)

and therefore we define the supremum of the rate

$$\Rightarrow r_{I,\mathrm{Supremum}}=\frac{\delta S}{N}$$ (17)

*r*_*I*,*Supremum*_ will substantially overestimate infectious spread because it applies the relatively high singleton death rate to all clones—clones with few cells become extinct more quickly than clones with many cells. To obtain a tighter upper bound we divide clones into those that are smaller and larger than an arbitrary size *f*_*max*_ and expand the expression for *r*_*I*_ in Eq (17) to obtain

$$r_{I,f_{\max}}=\frac{{\hat{\delta }}_{small}\sum_{f=1}^{f_{\max}}n_{f}+{\hat{\delta }}_{l\arg e}\sum_{f=f_{\max}+1}^{\infty }n_{f}}{N}$$ (18)

where *n*_*f*_ denotes the number of clones of frequency *f*, i.e. the "occupancy classes". The aggregate clone death rate of small clones ${\hat{\delta }}_{small}$ and of large clones ${\hat{\delta }}_{l\arg e}$ will comprise a weighted average of the death rate of clones of all sizes within that category. Because the HTLV-1 clonal frequency distribution is heavy tailed, small clones are more numerous than large clones, and so will make the dominant contribution to the clone death rate. Therefore the contribution from large clones can be neglected to give

$$r_{I,f_{\max}}≃\frac{{\hat{\delta }}_{small}\sum_{f=1}^{f_{\max}}n_{f}}{N}$$ (19)

Provided *f*_*max*_ is sufficiently small, then ${\hat{\delta }}_{small}$ (which is less than or equal to δ) can be approximated by δ. The error incurred by this approximation decreases as *f*_*max*_ is reduced, and so the infectious spread rate will be best approximated by $r_{I,f_{\max}}$ for low values of *f*_max_. Estimates of the ratio of infectious spread to mitotic spread can be obtained by dividing *r*_*I*,*Supremum*_ and $r_{I,f_{\max}}$ by the per-capita rate of mitotic spread *π =* 0.0316 (S1 Text) to give

$$R_{Supremum}=r_{I,Supremum}/\pi$$ (20)

and

$$R_{f}{}_{\max}=r_{I,f_{\max}}/\pi .$$ (21)

### Modelling approach 3: Occupancy class model

**Fig 4:**
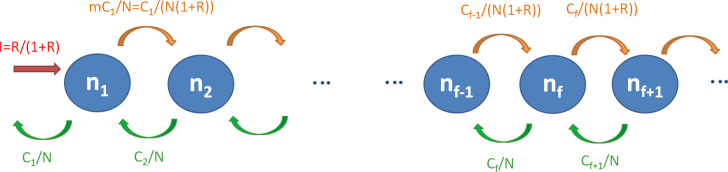
Occupancy class model schematic. Singletons (clones of size 1) are produced by infectious spread (red). Proliferation (orange) results in loss from clone size class *n*_*f*_ and entry into size class *n*_*f +* 1_. Death of a cell (green) results in a clone moving from size class f to size class *n*_*f—*1_.

Adapting a model of naïve T cell dynamics [46], we model the occupancy classes *n*_*f*_ of HTLV-1 clones (Fig 4). We assume that the clonal structure is in equilibrium (i.e. that the number of clones in each size class is constant) and that the probabilities of cell proliferation and death are independent of clone size.

Scaling so there is one event (i.e. de novo infection or mitosis) per cell per unit time we have *I + M = 1* and *R*: *= I / M*. Therefore

I=R/(1+R) (22)

and

M=1/(1+R) (23)

where *I* and *M* are the rates of infectious and mitotic spread (scaled as above), and *R* is the ratio of infectious to mitotic spread.

A clone in occupancy class *f* moves to class *f+*1 by mitosis with probability

$$Mfn_{f}/N=fn_{f}/N\left(1+R\right)$$ (24)

where *N* is the number of infected cells. A clone in occupancy class *f+*1 moves down to class *f* by death. Loss of cells by death is equal to the production of new cells by infection and mitosis, which has been scaled to 1, so the death rate is 1 per unit time. Since we assume that the probability of death is independent of clone size, the probability that the one death event in unit time occurs to a cell in size class *i+1* is simply equal to the proportion of cells in size class *i+1* i.e. *fn*_*f*_ */ N*.

In order for the number of cells *C*_*f*_ in size class *f* (*C*_*f*_ = *fn*_*f*_) to remain constant, we require that flow in and flow out of the occupancy class *n*_*f*_ to be equal (Fig 4), i.e. that the number of cells leaving occupancy class *n*_*f*_ must be equal to those arriving from class *n*_*f-1*_ (via mitosis) and class *n*_*f+1*_ (via cell death). By reference to Fig 4 we see that, for clone class f, we therefore have

$$\frac{1}{1+R}\frac{C_{f-1}}{N}+\frac{C_{f+1}}{N}=\frac{1}{1+R}\frac{C_{f}}{N}+\frac{C_{f}}{N}\;\mathrm{for}\;f=2,\ldots ,\infty$$ (25)

Rearranging gives

$$C_{f+1}=\left(\frac{1}{1+R}+1\right)C_{f}-\frac{1}{1+R}C_{f-1}$$ (26)

For the number of cells (*C*_*1*_) in size class 1 to remain constant we require

$$\frac{R}{1+R}+\frac{C_{2}}{N}=\frac{1}{1+R}\frac{C_{1}}{N}+\frac{C_{1}}{N}$$ (27)

And for the population as a whole to remain of constant size we need the gain of new clones to balance their loss

$$\frac{R}{1+R}=\frac{C_{1}}{N}$$ (28)

Rearranging (28) gives our first estimator (*R*_*1*_) for the ratio *R* from the occupancy class model, given in terms of *p = C*_*1*_*/N*, the proportion of cells that are singletons:

$$R=\frac{p}{1-p}$$ (29)

Substituting (28) into (27), and applying (26) recursively we obtain

$$C_{f}=\frac{1}{1+R}C_{f-1}\;\mathrm{for}\;f=2,3\ldots \infty$$ (30)

Specifically, substituting (28) into (27), replacing R/1+R we obtain

$$\begin{array}{l} \frac{C_{1}}{N}+\frac{C_{2}}{N}=\frac{1}{1+R}\frac{C_{1}}{N}+\frac{C_{1}}{N} \end{array}$$

giving

$$\begin{array}{l} C_{2}=\frac{1}{1+R}C_{1} \end{array}$$

i.e. (30) when *f* = 2.

To get a similar expression for *C*_*3*_ we replace *f* = 3 in (26) to give

$$C_{3}=\left(\frac{1}{1+R}+1\right)C_{2}-\frac{1}{1+R}C_{1}$$

Eliminating *C*_*1*_

$$\begin{array}{l} C_{3}=\left(\frac{1}{1+R}+1\right)C_{2}-C_{2}\\ C_{3}=\frac{1}{1+R}C_{2} \end{array}$$

i.e. (30) when *f* = 3 and so on, recursively to obtain the general Eq (30) by inspection.

And thus

$$C_{f}={\left(\frac{1}{1+R}\right)}^{f-1}N\frac{R}{1+R}.$$ (31)

Species richness is defined as the number of clones, and so

$$\begin{array}{l} \mathrm{Species}\;\mathrm{richness}=\sum_{f=1}^{\infty }n_{f}\\ \;=\sum_{f=1}^{\infty }\frac{C_{f}}{f}\\ \;=\sum_{f=1}^{\infty }{\left(\frac{1}{1+R}\right)}^{f-1}\frac{N}{f}\frac{R}{1+R} \end{array}$$ (32)

obtained by substituting in (31).

Using the fact that $\sum_{k=1}^{\infty }\frac{z^{k}}{k}=\ln\left(\frac{1}{1-z}\right)$ (a special case of the polylogarithm function)

We have that

$$\mathrm{species}\;\mathrm{richness}=\ln\left(\frac{1+R}{R}\right)NR$$ (33)

Rearranging for *R*, this provides our second estimator for the ratio of infectious to mitotic spread, *R*_*2*_, from the occupancy class model.

The number of infected cells in the body is estimated from each patient’s proviral load, as described in [10]. The proportion of infected cells that are singletons is estimated using the DivE distribution generation algorithm, and given in S1 Table. This algorithm estimates the relative frequencies of observed and unobserved clones from extrapolated rarefaction curves. Further details are given in [10].

We have outlined three methods: a deterministic and stochastic hybrid model of individual HTLV-1 clones over time; a greatly simplified model to approximate the upper bound of the infectious spread rate; and a model of clone occupancy classes. This final method yields two estimators.

## Results

### HTLV-1 clonal diversity estimates

We previously estimated HTLV-1 clonal diversity (the number of unique clones) from sequence data in 11 subjects with non-malignant HTLV-1 infection (either asymptomatic carriers, HTLV-1-associated uveitis patients or those with HAM/TSP). These estimates were obtained by measuring diversity in the nine blood samples per person (three at each of three time points) and then applying our recently developed method of estimating clonal diversity by extrapolation from the sample to the whole body [10] (Table 1, S1 Table and S1 Fig).

**Table 1:** Hybrid model estimates of rate of infectious spread estimates and ratio of infectious to mitotic spread by patient.

Patient (Disease Status^‡^)	Mean Proviral load* (no. HTLV-1^+^ cells per 10,000 PBMCs) [9]	Mean Estimated* diversity (no. HTLV-1^+^ clones in body) [10]	Infectious spread rate *r*_*I*_ (d^-1^) [Mean, (Lower–Upper)^†^, standard deviation within patient replicate samples]	Ratio of infectious to mitotic spread [Mean, (Lower–Upper)^†^, standard deviation within patient replicate samples]	Number new clones per day [Mean, (Lower–Upper)^†^],
1 (AC)	417	50666	1.0e-09, (5.9e-10–1.4e-09), 2.6e-10	3.3e-08, (1.9e-08–4.4e-08), 8.3e-9	149, (101–191)
2 (UV)	133	19025	1.1e-09, (4.8e-10–1.6e-09), 3.5e-10	3.5e-08, (1.5e-08–5.0e-08), 1.1e-8	51, (25–67)
3 (HAM)	320	59908	1.7e-09, (1.2e-09–2.1e-09), 3.0e-10	5.2e-08, (3.9e-08–6.8e-08), 9.6e-9	181, (130–243)
4 (HAM)	920	36840	2.8e-10, (2.1e-10–3.7e-10), 5.4e-11	8.8e-09, (6.8e-09–1.2e-08), 1.7-.9	89, (68–113)
5 (HAM)	160	16485	7.8e-10, (5.2e-10–1.0e-09), 1.9e-10	2.5e-08, (1.6e-08–3.3e-08), 6.0e-9	43, (33–58)
6 (HAM)	187	15906	6.1e-10, (3.4e-10–9.5e-10), 2.3e-10	1.9e-08, (1.1e-08–3.0e-08), 7.3e-9	39, (19–57)
7 (HAM)	2077	152180	5.9e-10, (4.9e-10–6.8e-10), 6.7e-11	1.9e-08, (1.5e-08–2.2e-08), 2.1e-9	428, (346–496)
8 (HAM)	1753	52246	2.1e-10, (1.6e-10–3.3e-10), 5.9e-11	6.8e-09, (4.9e-09–1.0e-08), 1.9e-9	128, (82–178)
9 (HAM)	1827	142032	7.3e-10, (5.3e-10–1.1e-09), 2.2e-10	2.3e-08, (1.7e-08–3.4e-08), 6.9e-9	456, (303–671)
10 (HAM)	813	68897	6.8e-10, (6.1e-10–7.6e-10), 6.4e-11	2.2e-08, (1.9e-08–2.4e-08), 2.0e-9	196, (157–249)
11 (HAM)	690	59145	7.6e-10, (4.2e-10–1.6e-09), 4.1e-10	2.4e-08, (1.3e-08–4.9e-08), 1.3e-8	161, (118–234)
**Mean**	845	61212	7.7e-10 d^-1^	2.4e-8	175

* Mean value of nine replicate samples for each patient (see methods)

^†^ Lower and Upper denote the range of estimates from nine hybrid model fits from each subject.

^‡^ Disease status: AC = asymptomatic carrier. UV = uveitis (non-HAM/TSP); HAM = HAM/TSP

We tested our assumption that the number of clones is at equilibrium in the chronic phase of infection, where HTLV-1 proviral load is at equilibrium. Using a two-tailed binomial test, we found little evidence that this change was significantly different from zero (p = 1 for observed and p = 0.07 for estimated). We further used linear regression to estimate the net change per day in the observed and estimated number of clones in each subject. This net change was 0.01 (95% CI -0.07–0.09) clones per day (i.e. 1 clone every 100 days) and -2.50 (-5.94–0.93) clones per day in the observed and estimated number of clones respectively; in each case the confidence interval spans zero. We therefore make the approximation that HTLV-1 clonal diversity remains unchanged in the chronic phase of infection, after the proviral load has reached steady state.

It is important to note that this assumption requires some infectious spread to replenish dying clones. However, if there were no infectious spread, and given the cell death rate *δ* (0.0316 d^-1^) and the estimated number of singletons *n*_*1*_ (5.5 *×* 10^2^–2.3 *×* 10^4^, (S1 Table)), we would expect approximately *n*_*1*_ *× δ* clones to be lost every day, i.e. an average of 172 (between 18 and 718). This number is vastly more than we estimate. Furthermore, this logic is conservative as it ignores the number of clones with two cells, number of clones with three cells, etc. that will die. Finally, if there was no replacement of clones through infectious spread during chronic infection, we would expect the estimates of clonal diversity to decline within patients over time.

### Modelling approach 1: Full simulation hybrid model

We model within-host HTLV-1 persistence by considering HTLV-1-infected clones separately. Large clones are modelled deterministically using a system of ordinary differential equations. Smaller clones are modelled stochastically by solving the chemical master equation (Eqs (9) and (10)) that considers the frequency of each clone as a random variable governed by a birth-death process (Fig 2B). The per-capita rate of infectious spread and the expected number of infected cells are then combined to model the birth of new clones (Eq (11)), and the extinction probability of each clone is used to calculate the expected clone death (Eq (12)). The birth and death (extinction) of clones provide an estimate of the number of clones at equilibrium (Eq (13), Fig 2C), and it is this value that is fitted to our estimates of HTLV-1 clonal diversity, to infer the per-capita rate of infectious spread (Fig 2D).

**Fig 5:**
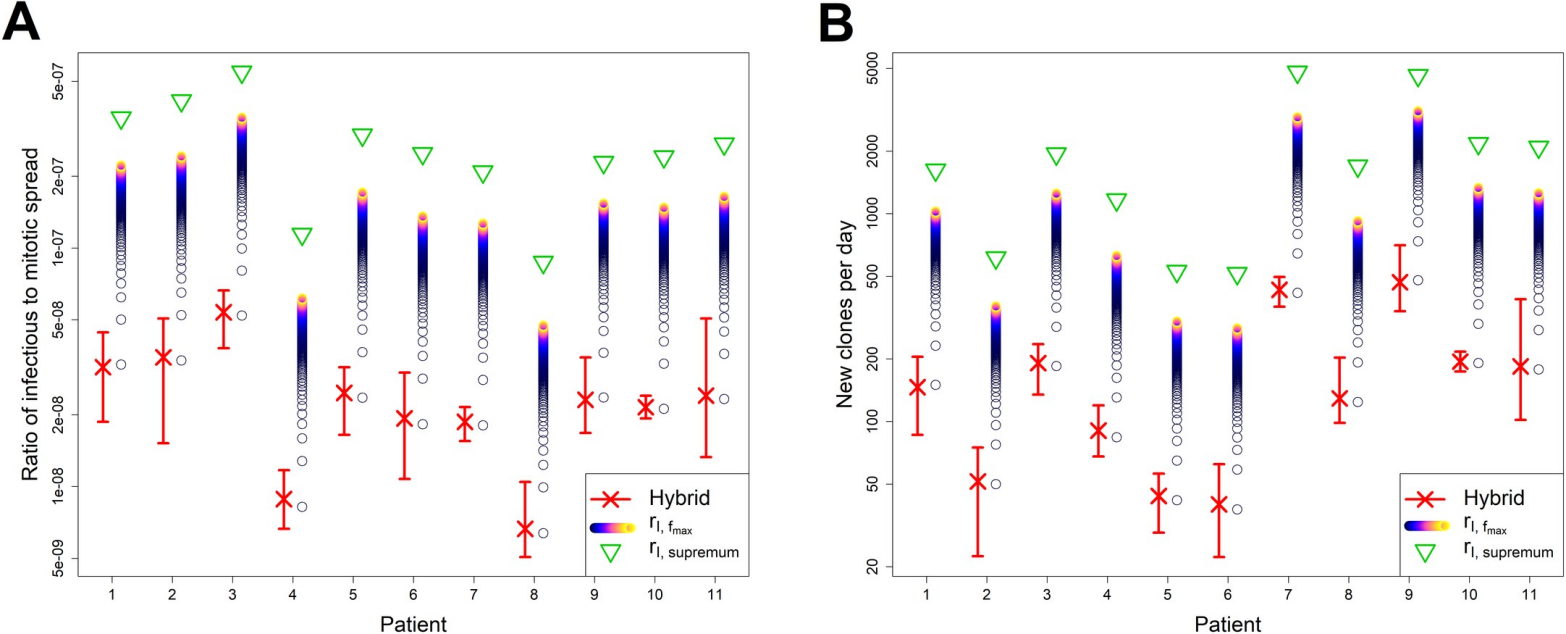
Ratio of infectious spread to mitotic spread and number of new clones per day, by patient and estimator. **A** Ratio of infectious spread to mitotic spread. **B** Number of new clones generated per day. Values of both the ratio and number of new clones are derived from estimators of infectious spread. In each plot, red crosses and bars respectively denote point estimates and the range from the nine estimates for each subject from the hybrid model. Upper bound approximations from *r*_*I*,*Supremum*_ (green triangles) are shown, together with tighter upper bounds from $r_{I,f_{\max}}$ (coloured circles) for multiple values of *f*_max_ between 1 and 1000. Lighter colours denote higher values of *f*_max_. Hybrid model point estimates are very close to the estimates obtained for *f*_max_ = 1 (lowest circles). Estimates plotted on logarithmic scale.

The hybrid model was fitted for each subject, for all samples at each time point, providing an estimate of the infectious spread rate in each case (Table 1, S3 Table). These nine estimates per patient were averaged to calculate the mean rate for each individual. Between individuals, the mean estimated rate of infectious spread was 7.7 × 10^−10^ per day, ranging from 2.1 × 10^−10^ to 1.7 × 10^−9^ per day (Table 1). Given an estimate of the rate of mitotic spread of 3.2 × 10^−2^ per day (Table 2), our infectious spread estimates imply an average ratio of infectious to mitotic spread of 2.4 × 10^−8^ (6.6 × 10^−9^–5.3 × 10^−8^), i.e. varying by almost an order of magnitude (Table 1, Fig 5A).

**Table 2:** Parameter names and values.

Parameter Name	Description	Comments	Value
*r*_*I*_	daily per-capita rate of infectious spread (de novo infection)	Fitted for each patient [Methods]	See Table 1
*π*	daily per-capita rate of mitotic spread (infected cell proliferation)	Derived from [35] (supplementary information)	0.0316 per day
*δ*	daily per-capita rate of infected cell death	Derived from [35] (supplementary information)	0.0316 per day
*K*	Density dependency parameter. Infected cell proliferation rates are half maximal when number of infected cells *N(t) = K*	Derived from [35] (supplementary information)	4.02 ×10^11^
*R*	Ratio of infectious to mitotic spread	derived from value of *π* and fitted values of *r*_*I*_	See Table 1

While the per-capita infectious spread rate is very low, it translates to an average of 175 (range 39–456) new clones created per day (Fig 5B). Therefore the hybrid model predicts that infectious spread is not limited to initial infection, but persists at a low level throughout the chronic phase. Over a 10-year period, this amounts to an average of 6.4 × 10^5^ clones that are created and destroyed (1.4 × 10^5^–1.7 × 10^6^). As each clone will integrate in a new genomic site, low but sustained levels of infectious spread could be an alternative mechanism by which chronic infection develops into malignancy.

Within individuals the standard deviation between samples in the infectious spread rate was relatively small, with an average of 2 × 10^−10^ (5.4 × 10^−11^–4.1 × 10^−10^) (Table 1). Estimates of the per-capita infectious spread rate were not found to correlate with either proviral load or with the estimated diversity during the chronic phase (this may be due to our 11 patients providing insufficient power). However, unsurprisingly, the estimated number of new clones per day was correlated with both proviral load (R^2^ = 0.62) and strongly correlated with the estimated diversity (R^2^ = 0.99) (S2 Fig).

### Sensitivity analyses of hybrid model

We investigated the sensitivity of our results to: i) choice of threshold frequency *F* (above and below which clones are respectively modelled deterministically and stochastically); ii) choice of time step; iii) use of hybrid deterministic and stochastic model (as opposed to a purely stochastic model); and iv) choice of proliferation and death parameters.

Originally our threshold value of *F* was set to equal 100. However, the extinction probability of clones of size 100 over a duration of *t*_*Dur*_ = 3133 days (S2 Text) duration was 0.37. We were therefore concerned that excluding such clones would bias the estimates of the infectious spread rate and therefore the ratio, and so re-fitted our model with *F* = 460. This value is the minimum clone frequency for which the extinction probability is less than 1%, given our parameters of infected cell growth, death, and density dependency (S3 Fig, S2 Text). The estimates of infectious spread from the hybrid model are almost identical whether we assume *F* = 100 or *F* = 460. We present the *F* = 460 estimates, as the most accurate description of the system would to consider all clones stochastically.

We find that a timestep of *h* = 1 day results in negligible splitting errors (S4 Fig), and that using a hybrid deterministic and stochastic system gives an appropriate approximation of our stochastic process (S5 Fig).

We investigated the sensitivity of the hybrid to proliferation and death rates. We fitted the model again, assuming these rates were halved (to give π* = δ = 0.0158) or doubled (to give π* = δ = 0.0632), while still modelling proviral load at equilibrium. We find that the ratio of infectious spread to mitotic spread is robust to these different values, giving almost identical results. However, the infectious spread rate *r*_*I*_, and therefore the number of new clones per day are sensitive to proliferation, death, and density-dependency parameters *π*, *δ* and *K* (S3 Table, S6 Fig), although results are similar. When rates were halved and doubled, the average number of new clones per day was respectively 89 (20–234) and 341 (77–885).

Although the population is still at equilibrium, higher proliferation and death rates cause the birth-death process for each clone to behave more “erratically”, thus increasing the probability of clone extinction, and the ensuing higher clone death must be met with additional infectious spread.

### Modelling approach 2: Upper bound approximation

Upper bounds of the infectious spread rate (*r*_*I*,*Supremum*_) were estimated for each subject using Eq (17), by substituting inputs of HTLV-1 clonal diversity estimates (Table 1, S1 Table) and an estimate of *δ* = 0.0316 infected cell death a day, and an estimate of the total number of infected cells *N* (derived from the proviral load, as detailed in [10]). For each individual we averaged across all samples and across all time points. Estimated values of the rate ranged between individuals from 2.8 × 10^−9^ to 1.7 × 10^−8^ per infected cell per day, and thus (given a per-capita rate of mitotic spread of 0.0316 cells per day) estimates of the ratio *R*_*Supremum*_ ranged between 8.7 × 10^−8^ and 5.5 × 10^−7^ (Fig 5A). The estimated number of new clones per day using *r*_*I*,*Supremum*_ are unsurprisingly much larger than those of the hybrid, ranging from 516 to 4804, i.e. approximately an order of magnitude higher (Fig 5B).

We further estimated the more restrictive upper bounds of the ratio $R_{f_{\max}}$ from Eq (21) for multiple *f*_max_ values between 1 and 1000 (Fig 5A). These estimates assume that the cell death rate applies to clones with frequencies less than or equal to *f*_max_, and that larger clones do not contribute to the rate.

The hybrid estimates always fall below the estimated supremum and are very close to the estimates provided by for *f*_max_ *=* 1 (Fig 5, S6 Fig). Since it is likely that the upper bound approximation will give more accurate estimates for lower values of *f*_max_, this result demonstrates the consistency of estimates produced between the hybrid and the upper bound approximation, and further implies that the number of singletons is the dominant factor in the extent of infectious spread. We estimate that singletons constitute between 6% and 12% of clones (S1 Table).

### Modelling approach 3: Occupancy class model

The results from the hybrid model indicate a very low ratio of infectious to mitotic spread. The hybrid benefits from treating small clones stochastically and from the inclusion of known experimental details of HTLV-1 infection and spread. However, it remained possible that these very low estimates of the ratio resulted from incorrect model or parameter assumptions. To test the robustness of our estimate of the ratio to changes in model and parameter assumptions, we adapted a simple deterministic model of HTLV-1 clonal dynamics and occupancy classes and used this to produce two alternative estimators of the ratio of infectious to mitotic spread.

The occupancy class model is based on a model of naïve T cell dynamics developed by de Greef et al [46]. It assumes that clonal dynamics are deterministic, that the clonal structure is in equilibrium and that the probabilities of cell proliferation and death are independent of clone size. The model yields two estimators of the ratio of infectious to mitotic spread. The first estimator (referred to as *R*_*1*_) depends on the proportion of infected cells that are singletons

$$R_{1}=\frac{p}{1-p}$$

where *p* is the proportion of cells that are singletons.

The second estimator (referred to as *R*_*2*_) depends on species richness.

$$\mathrm{species}\;\mathrm{richness}=\ln\left[\frac{1+R_{2}}{R_{2}}\right]NR_{2}$$

where *N* is the number of infected cells (see Methods for derivation of both expressions).

**Fig 6:**
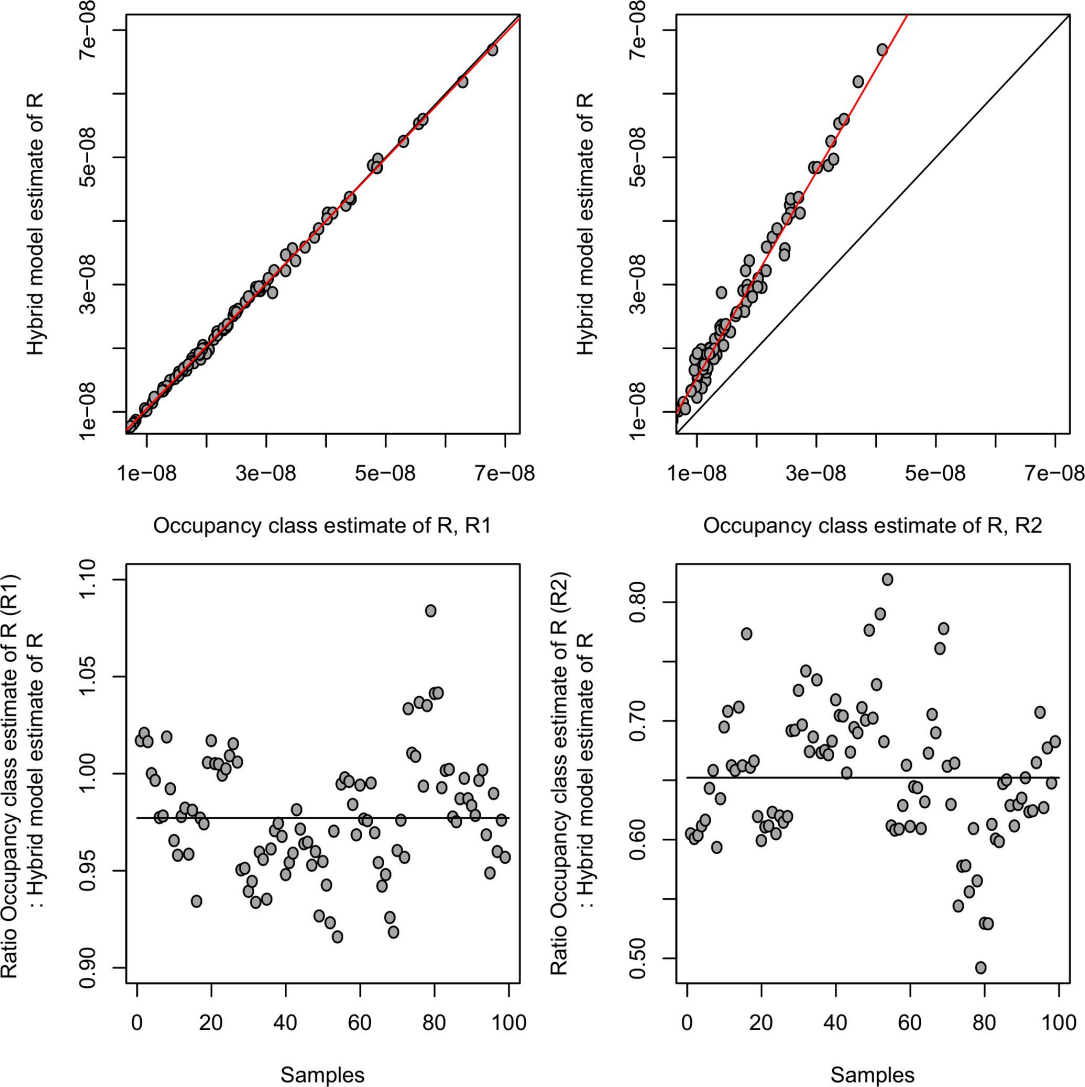
Comparison of estimates of ratio of infectious to mitotic spread from the hybrid model (method 1) and the occupancy class model (method 3). (Top left) Estimate of ratio from hybrid model plotted against first estimate from occupancy class model (*R*_*1*_). Red line is line of best fit, black line is line of equality. (Top right) Estimate of ratio from hybrid model plotted against second estimate from occupancy class model (*R*_*2*_). Red line is line of best fit, black line is line of equality. (Bottom left) Estimate of ratio between hybrid model and first estimate from occupancy class model (*R*_*1*_). Black line denotes the median. (Bottom right) Estimate of ratio between hybrid model and second estimate from occupancy class model (*R*_*2*_). Black line denotes the median.

Across the 99 estimates (11 subjects, 3 time points, 3 replicates) both estimators, *R*_*1*_ and *R*_*2*_, are strongly positively correlated with the estimate of the ratio produced by the hybrid model (P = 1 × 10^−135^ and P = 6 × 10^−87^ respectively, Pearson correlation) and agree well numerically, being of the same order of magnitude and, if anything tending to be even smaller (hybrid median = 2.0 × 10^−8^, hybrid LQ = 1.4 × 10^−8^, hybrid UQ = 3.0 × 10^−8^; *R*_*1*_ median = 2.0 × 10^−8^, *R*_*1*_ LQ = 1.4 × 10^−8^, *R*_*1*_ UQ = 3.0 × 10^−8^; *R*_*2*_ median = 1.3 × 10^−8^, *R*_*2*_ LQ = 1.0 × 10^−8^, *R*_*2*_ UQ = 1.9 × 10^−8^) (Fig 6).

Finally, we applied the second estimator from the occupancy class model (*R*_*2*_) to the Chao1 estimator of clonal diversity (rather than the DivE estimate used up to this point). The Chao1 estimator gives much lower diversity estimates, and so unsurprisingly yields considerably smaller estimates of the infectious to mitotic spread ratio (median = 7.3 × 10^−10^, LQ = 4.7 × 10^−10^, UQ = 1.0 × 10^−9^).

We conclude that the low estimates of the infectious to mitotic spread are not the product of implicit assumptions in the hybrid model or incorrect parameter choice. Inaccurate estimates of the clonal diversity may play a significant role but calculations using an alternative, widely used estimator provided even smaller estimates of clonal diversity, and therefore yield an even lower ratio. The lower estimates of the ratio from Chao estimator are driven by its lower diversity estimates. However we found previously that these diversity estimates were unrealistically low, in that they predicted fewer clones than were observed in additional blood samples taken from the same subject at the same time, whereas DivE did not [10]. Our estimates of ongoing infectious spread during chronic infection are therefore more likely to be an underestimate than an overestimate.

## Discussion

The relative contribution of infectious and mitotic spread to HTLV-1 viral persistence has not previously been estimated, and this has been a long-standing problem in the field. For many years, it was believed that the virus persisted solely by oligoclonal proliferation of latently infected cells, and that infectious spread contributed little if anything to persistence. However, three observations have brought this belief into question. First, the strong, persistently activated host T-cell response to HTLV-1 implied that the virus is not latent but is frequently expressed in vivo. Second, high-throughput analysis revealed that a typical host carries between 10^4^ and 10^5^ clones, not ~100 clones as was previously believed. Third, treatment with the antiretroviral therapy lamivudine temporarily but substantially reduced the proviral load of a patient with HAM/TSP. These observations raise the question: what is the contribution of infectious spread to the persistence of HTLV-1 in the host after initial infection?

In this study, we used three different strategies to estimate the ratio of infectious to mitotic spread during the chronic phase of infection. We first developed a deterministic and stochastic hybrid model of within-host HTLV-1 dynamics, and fitted this model to clonal diversity estimates derived from experimental data. We then derived an estimate of the upper bound of the ratio by using a highly simplified model where individual clones are not considered. Finally, we adapted a model of naïve T cell repertoires that models clone occupancy classes. We found broad agreement between the estimates of the ratio obtained using all three methods; and each method implied the existence of ongoing infectious spread during chronic infection, after the HTLV-1 proviral load has reached steady state.

We found no evidence that HTLV-1 clonal diversity either increases or decreases over time, and we therefore assumed that the number of HTLV-1 clones is in equilibrium during chronic infection. It is important to note that this assumption necessitates some infectious spread by design. However, the estimated number of clones changes far less over time than would be expected if there were no infectious spread, given the cell death rate and proportion of clones that are singletons, and so we believe this assumption is justified.

While the ratio of infectious to mitotic spread during the chronic phase is very small (~2 × 10^−8^), it equates to ~10^2^ new clones every day. That is, approximately 100 new HTLV-1-infected T cell clones appear every day by infectious spread. Over a 10-year period, this would result in an average of 6.4 × 10^5^ clones created and subsequently destroyed. Further, while the estimated rate of infectious spread represents a negligible contribution to HTLV-1 proviral load, the constant creation of new clones will increase the risk of malignant transformation, because this risk depends in part on the proviral integration site [21]. Malignancy could arise not only from accumulated mutations in a long-lived clone (where only mitotic spread is relevant), but also from frequent proviral integration (where only infectious spread is relevant).

High HTLV-1 proviral load increases both clonal diversity [47] and risk of ATL [7]. However, it is unknown whether the increased clonal diversity (caused by infectious spread) is a mechanism for this higher risk of malignancy, or whether it is a separate by-product of high proviral load. Ongoing infectious spread during chronic infection would be consistent with the hypothesis that higher infectious spread increases the risk of malignant transformation. If this is the case, then anti-retroviral therapy could reduce the risk of ATL in patients who have entered their chronic phase, not by sustainably suppressing proviral load, but by limiting proviral integration; this therapy would need to be continued for many years before its impact was evident.

It is important to note that the different methods we use are not independent. First, they all use our clonal diversity estimates as an input (see section below). Second, they all assume equilibrium clonal diversity. However, they do differ in a number of respects. The upper bound approximation is independent of the parameters *F*, *π* and *K* and makes no assumptions about the clonal structure or the density dependence of infected cell proliferation. The *R*_*1*_ estimator from the occupancy class model depends only on the proportion of singletons and so is independent of all the parameters (*F*, *π*, *δ* and *K*), assumptions about density dependence of proliferation, and indeed the estimated clonal structure beyond the number of singletons. Similarly the *R*_*2*_ estimator from the occupancy class model is also independent of *F*, *π*, *δ* and *K* as well as proliferation assumptions. While the hybrid model is our most detailed simulation of HTLV-1 within-host dynamics, it is mathematically and computationally complex and requires significant runtime. Because the estimates from all three methods are largely consistent, our analysis indicates that the latter two methods provide good approximations of the rate of infectious spread and the ratio of infectious to mitotic spread.

The most likely source of error in our estimates of the ratio of infectious to mitotic spread lies in the estimation of clonal diversity. Two factors argue against a serious error. First, estimates based on two different quantities (the number of clones and the proportion of infected cells that are singletons) give very similar estimates of the ratio. Second, diversity estimates from DivE are far more plausible than those from other widely used estimators of species richness: these other estimators produced demonstrably false estimates that were lower than the diversity observed by combining blood independent samples drawn at the same time from the same patient. [10]. It remains possible that we have underestimated clonal diversity, although if so then our conclusion of ongoing infectious spread during chronic infection would only be strengthened.

A much smaller source of potential error lies in using the number of clones to quantify infectious spread. If the virus repeatedly integrates in the same genomic site, then the number of unique genomic sites would be less than the number of true clones, and hence both the infectious spread rate and the ratio would be underestimated. However, hotspots of HTLV-1 integration have not been observed [9], and so such repeat infection would not substantially alter our estimate. Assuming the provirus does not efficiently integrate into heterochromatin, which represents ~2/3 of the human genome, then only one third of the ~3 × 10^9^ base pairs of the human genome have the potential for proviral integration. The probability of repeated proviral integration is then the number of existing integration sites divided by the number of potential integration sites. Given the estimated number of clones is of the order of 10^5^, this probability is approximately 10^5^/10^9^ = 10^−4^. Therefore, any error in using the number of clones to quantify infectious spread infectious spread is very small.

It seems surprising that, during initial infection, the virus could establish a stable population of infected T cell clones with such a low rate of infectious spread. However, these low rates of infectious spread are measured in the chronic phase of infection, when the strong host cytotoxic response kills HTLV-1-expressing cells, which probably reduces efficient infectious transmission and favours mitotic transmission. During the early phase of infection, before the establishment of an adaptive immune response, the contribution of infectious spread is likely to be substantially higher than during chronic infection. It would be interesting to model the dynamics of early infection, in particular to investigate the rate required to establish a stable population of infected T cell clones. Modelling early infection would violate the assumption of equilibrium, and thus would void many of the simplifying assumptions that makes our model tractable (e.g. our ability to model clones independently and so avoid an exponential increase in complexity). However, given sufficient computational power, this analysis would be possible.

Another extension of each of the methods presented might be to include variable proliferation rates for each clone, which would refine estimates of the infectious spread rate and ratio of infectious spread to mitotic spread. Because clones proliferate in response to antigen, our assumption of a single per-capita infected cell proliferation rate is a simplifying approximation. If larger clones are larger in part because they proliferate faster than small clones, then small clones will die at a faster rate, which would likely require more infectious spread to maintain equilibrium of clonal diversity.

The methods described here have potential applications in other fields, for example in modelling the human T cell receptor (TCR) repertoire. The mechanisms by which the immune system is reconstituted after immune suppression or transplantation are poorly understood. Drawing parallels between immune reconstitution and HTLV-1 infectious and mitotic spread, the present approach could be applied to investigate the extent to which reconstitution occurs either through the generation of new TCR clonotypes, or through the expansion of existing clonotypes. In HIV-1 infection, the approach could be used to quantify the ratio of infectious to mitotic spread in the absence of treatment and in the latent reservoir remaining following treatment.

In summary, we develop three methods, which have the potential to be applied to a range of areas, and use them to quantify the role of de novo infection in HTLV-1 persistence during chronic infection. We find that on average 5 x 10^9^ new infected cells are produced every day; of these the vast majority (>99.9%) will arise from division of an existing infected cell and will thus have the same proviral integration site as their mother cell, but a small minority (about 175 cells per day) will arise from infectious transmission and will contain a novel proviral integration site. These estimates suggest that ongoing infectious spread may be a mechanism for malignant transformation that treatment with antiretroviral drugs may suppress.

## Supporting information

S1 Text Estimation of density dependency parameters. (PDF)

S2 Text Additional details of hybrid model. (PDF)

S3 Text Further hybrid model sensitivity analysis. (PDF)

S1 Table Patient sample characteristics and diversity estimates. (PDF)

S2 Table Notation used in models of HTLV-1 within host persistence. (PDF)

S3 Table Hybrid model sensitivity to proliferation and death rates. (PDF)

S1 Fig Observed and estimated diversity (number of HTLV-1^+^ clones) over time by patient. Estimated diversity is shown in blue (left hand y-axes) and observed diversity is shown in red (right-hand y-axes).
(TIF)

S2 Fig Correlations between per-capita infectious spread rate, daily number of new clones, estimated clonal diversity and HTLV-1 proviral load. Infectious spread rate is not correlated with either the estimated diversity (**A**, R^2^ = 0.0069) during the chronic phase or proviral load (**B**, R^2^ = 0.28). However, unsurprisingly, the estimated number of new clones per day was strongly correlated with both the estimated diversity (**C**, R^2^ = 0.99) during the chronic phase or proviral load (**D**, R^2^ = 0.62) and proviral load.
(TIF)

S3 Fig Choice of stochastic threshold frequency *F* and individual clone state space upper limit *τ* values for hybrid model. **A** Extinction probabilities by clone starting frequency (red) at *t*_*Dur*_ = 3133 days (given our values of infected cell proliferation and death parameters). Stochastic threshold frequency *F* = 460 (green) is chosen to be the minimum starting frequency such that a clone has less than a 1% (black line) chance of extinction. **B** Probability distribution of a clone at *t*_*Dur*_ = 3133 days given starting frequency *F =* 460. The upper limit *τ* (blue) is chosen so that this probability distribution is not significantly distorted.
(TIF)

S4 Fig Effect of hybrid time step length *h*. Expected diversity *S(t*_*Dur*_*)* at given duration (*t*_*Dur*_ *=* 600 days) (first row); minimum diversity predicted throughout given duration (second row); and the number of infected cells *N(t*_*Dur*_*)* predicted at *t*_*Dur*_ (third row), plotted against time step length *h* for three parameter sets: *θ*_*G*_ = {*r*_*I*_ = 2 × 10^−11^, π* = 0.0316, *δ* = 0.5 * π*} (left column—“growth”); *θ*_*E*_ = {*r*_*I*_ = 2 × 10^−11^, π* = 0.0316, *δ* = π*} (middle column—“equilibrium”); *θ*_*D*_ = {*r*_*I*_ = 2 × 10^−11^, π* = 0.0316, *δ* = 2 * π*} (right column—“death”) for an example patient data set. Note that y-axes are split so that time step differences are visible. Time step effects are negligible for each observable.
(TIF)

S5 Fig Comparison of pure stochastic and hybrid deterministic and stochastic model on reduced system. Reduced system omits all clones of size greater than 460 cells. For each patient, both the estimated number of clones over time and the fitted values of *r*_*I*_ are very similar between the pure stochastic and the deterministic/stochastic hybrid model.
(TIF)

S6 Fig Ratio of infectious spread to mitotic spread and number of new clones per day, by patient, estimator, and parameter choice. As per Fig 5. **A, C** Ratio of infectious spread to mitotic spread. **B, D** Number of new clones generated per day. **A, B** assume π* = δ = 0.0158, half the value in our main analysis. **C, D** assume π* = δ = 0.0632, twice the value in our main analysis. Estimates of the ratio of infectious spread to mitotic spread are almost identical. The infectious spread rate and therefore the number of new clones per day are sensitive to choice of proliferation and death parameters, although values are comparable. Upper bound approximations for *f*_*max*_ = 1 again match the more-detailed hybrid model.
(TIF)
