# Supplementary material for: The relative contributions of infectious and mitotic spread to HTLV-1 persistence
Source: PLoS Comput Biol. 2020 Sep 17;16(9):e1007470. doi: 10.1371/journal.pcbi.1007470 (PMC7524007; doi:10.1371/journal.pcbi.1007470)
Supplement: S1 Text — (PDF) [file pcbi.1007470.s001.pdf]

Estimation of π, δ and K

We describe the dynamics of all CD4$^+$CD45RO$^+$ cells y by

$$\dot{y} = \frac{p}{k+y} y - \mu y$$

the dynamics of uninfected CD4$^+$CD45RO$^+$ cells n by

$$\dot{n} = \frac{p'}{k+y} n - \mu' n$$

and the dynamics of infected cells x by

$$\dot{x} = \frac{\pi}{k+y} x - \delta x$$

where y is the total number of CD4$^+$CD45RO$^+$ T cells, x is the number of infected CD4$^+$CD45RO$^+$ T cells, n is the number of uninfected CD4$^+$CD45RO$^+$ T cells ($y = x + n$), p/k is the maximal proliferation rate of CD4$^+$CD45RO$^+$ cells (half maximal when $y = k$), p'/k is the maximal proliferation of uninfected CD4$^+$CD45RO$^+$ cells, π/k is the maximal proliferation rate of infected CD4$^+$CD45RO$^+$ cells and μ, μ' and δ the death rates of y, n and x respectively.

To estimate π and k we need estimates of the per capita proliferation rate $\pi / (k + y)$ at two values of y. We chose in a lymphocyte replete ($y = y_R$) and in a lymphocyte depleted host ($y = 0$)

$y = y_R$

We first estimate the per capita proliferation rate of infected CD4$^+$CD45RO$^+$ T cells

$$\pi^* = \frac{\pi}{k + y_R}$$

in a lymphocyte replete host.

The data provided in Supplementary Table 2A Asquith et al [16] gives the estimated proliferation rate of $CD4^+CD45RO^+$ T cells as a function of proviral load (measured as % of PBMC infected). If we convert proviral load to fraction of $CD4^+CD45RO^+$ T cells infected assuming that all proviral load is in $CD4^+CD45RO^+$ T cells and that $CD4^+CD45RO^+$ T cells make up approximately 15% of PBMC then we can plot the following relationship:

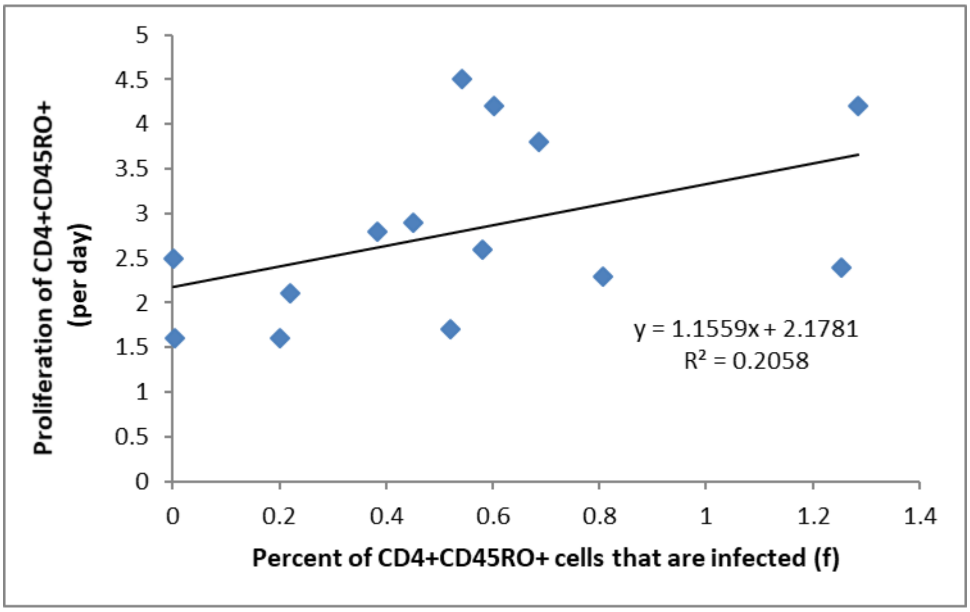

The measured proliferation rate in infected individuals is a combination of the proliferation rate of uninfected $CD4^+CD45RO^+$ cells $p^* / (k + y)$ at rate previously estimated to be approximately 2% per day [16-18] (consistent with the estimate of

2.18% per day above when $f = 0$) and the proliferation rate $\pi^* = \pi / (k + y)$ of infected cells

$$\text{proliferation of CD4+CD45RO+ cells} = 2(1-f) + \pi^* f$$

Comparing with the equation of a straight line above we can see that $\pi^* = 1.1559 + 2 = 3.16\ d^{-1}$

So in lymphocyte replete HTLV-1 infected host the average proliferation rate of infected cells is 3.16% per day. We can therefore conclude that at equilibrium δ = 3.16% per day.

$y = 0$

In a lymphocyte depleted host ($y = 0$) the proliferation rate will be maximal. We assume that this is 100% per day.

We estimate that the number of $CD4^+CD45RO^+$ T cells in a lymphocyte replete adult is approximately 5.25×10^{11} (2×10^{12} lymphocytes, of which 75% are $CD3^+$, of which 70% are $CD4^+$, of which 50% are RO^+). We thus have two equations:

$$3.16 = \frac{\pi}{k + 5.25 \times 10^{11}}$$

$$100 = \frac{\pi}{k}$$

then by solving these two equations simultaneously we find

$\pi = 1.7 \times 10^{12}$ cells per day

$k = 1.7 \times 10^{10}$ cells

If we assume that at equilibrium the number of uninfected cells (n) is constant, and let $K = k + n$, then we can write $k + y = k + n + x = K + x$. K is the density dependency parameter used in the hybrid model (Eq (1) in main text).

In a typical HTLV-1 infected individual proviral load is of the order of 1% PBMC. Using the figures above ($CD4^+CD45RO^+$ T cells make up approximately 15% of PBMC, number of $CD4^+CD45RO^+$ T cells in a lymphocyte replete adult is approximately 5.25×10^{11}) we can estimate the number of uninfected $CD4^+CD45RO^+$ T cells in the body (n) as:

% of PBMC infected = 1%

% of $CD4^+CD45RO^+$ infected = 1% / 0.15 = 6.7%

Number of $CD4^+CD45RO^+$ uninfected (n) = $0.933 \times 5.25 \times 10^{11} = 4.9 \times 10^{11}$

And so

$K = k + n = 1.7 \times 10^{10} + 4.9 \times 10^{11} = 5 \times 10^{11}$ cells

So to summarise we estimate:

$\delta = 3.16\%$ per day

$\pi = 1.7 \times 10^{12}$ cells per day

$K = 5.1 \times 10^{11}$ cells

These parameters are used in the hybrid model. We note that in the upper bound model we only use δ (i.e. our estimates are independent of values chose for π and K) and for the occupancy class model we do not use any of these parameters (i.e. our estimates are independent of δ, π and K).
