# Supplementary material for: The relative contributions of infectious and mitotic spread to HTLV-1 persistence
Source: PLoS Comput Biol. 2020 Sep 17;16(9):e1007470. doi: 10.1371/journal.pcbi.1007470 (PMC7524007; doi:10.1371/journal.pcbi.1007470)
Supplement: S2 Text — (PDF) [file pcbi.1007470.s002.pdf]

Stochastic Model of Within-Host HTLV-1 Dynamics

The following is based on [1, 2]. Consider a system with a discrete number of individuals, where each individual belongs to exactly one species or type. Assume that there are $S \in \mathbb{N}$ species $(\mathcal{S}_1, \mathcal{S}_2, ..., \mathcal{S}_S)$, and that species $\mathcal{S}_i$ has a frequency $X_i(t) \in \mathbb{N}_0 = \mathbb{N} \cup \{0\}$ at time *t*. Assume further that all individuals of the same species are identical. Then the vector $X(t) = (X_1(t), X_2(t), ..., X_S(t))^T \in \mathbb{N}_0^S$ is a random variable that describes the population of the system at time *t*. In our case, the individuals are HTLV-1 infected cells, and species are HTLV-1 infected clones (populations of cells identically infected with HTLV-1 with a common site of proviral integration in the host genome).

Suppose there are ρ_1, …, ρ_C possible reactions in the system, where a reaction ρ_c $(c \in \{1, ..., C\})$ is a mapping given by

$$\rho_c : \mathbb{N}_0^S \overset{g_c}{\rightarrow} \mathbb{N}_0^S \tag{S1}$$

$$\rho_c : \sum_{i=1}^{S} \kappa_{ci}^{\text{IN}} \mathcal{S}_i \overset{g_c}{\mapsto} \sum_{i=1}^{S} \kappa_{ci}^{\text{OUT}} \mathcal{S}_i \tag{S2}$$

$$\rho_c : \kappa_{c1}^{\text{IN}} \mathcal{S}_1 + ... + \kappa_{cS}^{\text{IN}} \mathcal{S}_S \overset{g_c}{\mapsto} \kappa_{c1}^{\text{OUT}} \mathcal{S}_1 + ... + \kappa_{cS}^{\text{OUT}} \mathcal{S}_S \tag{S3}$$

where $\kappa_{ci}^{\text{IN}}, \kappa_{ci}^{\text{OUT}} \in \mathbb{N}_0$ are the number of individuals of the ith species respectively required for and present after the cth reaction, and $g_c > 0$ is the reaction constant, which represents the average probability that a particular combination of $\sum_{i=1}^{S} \kappa_{ci}^{\text{IN}} \mathcal{S}_i$ individuals

will react according to reaction ρ_c in an infinitesimal time interval [3]. We define the stoichiometric vector ν_c as the difference to the state vector *X(t)* made by reaction *c*

$$\nu_c = \left(\kappa_{c1}^{\mathrm{OUT}} - \kappa_{c1}^{\mathrm{IN}}, \ldots, \kappa_{cS}^{\mathrm{OUT}} - \kappa_{cS}^{\mathrm{IN}}\right)^T \in \mathbb{Z}^S \qquad \text{(S4)}$$

We consider three types of reaction (namely cell death, mitosis, or infectious spread from each clone). It is important to note that, within a particular clone, there is no source inflow from frequency 0 to frequency 1 in our birth-death process. That is, we do not have a reaction ρ_j such that

$$\rho_j : * \xrightarrow{\lambda} \mathcal{X} \qquad \text{(S5)}$$

for some constant value λ, as a clone cannot proliferate once it has become extinct.

The propensity function α_c of reaction ρ_c is the reaction constant g_c multiplied by the number of different combinations of individuals required for reaction ρ_c, and is given by

$$\alpha_c(x) = g_c \prod_{i=1}^{S} \frac{x_i!}{(x_i - \kappa_{ci}^{\mathrm{IN}})!(\kappa_{ci}^{\mathrm{IN}})!} \qquad \text{(S6)}$$

for a potential state of the system $x = (x_1, x_2, \ldots, x_S)^T \in \mathbb{N}_0^S$ (i.e. x_i is the number of particles of species S_i). The evolution of the state vector *X(t)* is given by

$$X(t) = y_0 + \sum_{c=1}^{C} P_c\left(\int_0^t \alpha_c(X(s))ds\right)\nu_c \qquad \text{(S7)}$$

for initial $y_0 \in \mathbb{N}_0^S$. Eq (S7) states that the population *X(t)* at time t is equal to the initial population y_0 plus the sum over all reactions of the change ν_c to the population

induced by each reaction, multiplied by the (random) number of times the reaction has occurred. The number of times each reaction occurs is given by a Poisson distribution in the time interval [*0, t*] with expected value of $\int_0^t \alpha_c(X(s))ds$, i.e. a Poisson distribution $\mathcal{P}_c\left(\int_0^t \alpha_c(X(s))ds\right)$.

Master Equation: Mass-Action Rate Birth-Death Process

The probability distribution associated to the random variable $X(t) \in \mathbb{N}_0^{S_{\max}}$ in (S7) is given by $\mathbb{P}(X;t) = \mathbb{P}(X(t) = y \mid X(0) = y_0)$, where $y, y_0 \in \mathbb{N}_0^{S_{\max}}$. $\mathbb{P}(X;t)$ is a column vector where each entry is a probability associated to a potential state of the random variable. It can be shown [2, 4-6] that $\mathbb{P}(X;t)$ is the solution to the Chemical Master Equation

$$\frac{\partial \mathbb{P}(X = y;t)}{\partial t} = \sum_{c=1}^{C} \left(\alpha_c(y - \nu_c)\mathbb{P}(X = y - \nu_c;t) - \alpha_c(y)\mathbb{P}(X = y;t) \right) \qquad \text{(S8)}$$

We can interpret Eq (S8) as follows: the rate of change in the probability of the state taking value *X(t) = y* is the sum over all reactions of the probability of arriving at state *X(t) = y* via reaction ρ_c (having previously been in state $X(t) = y - \nu_c$) minus the probability of leaving state *X(t) = y* via reaction ρ_c.

Transition Matrix

If there is no upper limit on the frequency a given clone may take, the state space is given by $\Omega = \mathbb{N}_0$ (Fig 3A). If however a clone is bounded by a maximum frequency $\tau \in \mathbb{N}$, then the state space is given by $\Omega_\tau = \{0, ..., \tau\}$ (Fig 3B). We refer to Ω_τ as a

"truncated state space". Using Ω_τ , we can summarise Eq (S8) using multiple, simpler differential equations

$$\frac{d\mathbb{P}(X_i;t)}{dt} = A\mathbb{P}(X_i;t) \qquad \text{for } i = 1, \ldots, S_{max} \qquad \text{(S9)}$$

where A is a $\tau \times \tau$ transition matrix associated with the state space Ω_τ [7, 8]. A is a tridiagonal sparse matrix with all entries equal to zero except:

$$A_{f,f} = -f(\pi^* + \delta), \qquad 0 \leq f \leq \tau$$

$$A_{f,f+1} = (f+1)\delta, \qquad 0 \leq f \leq \tau - 1 \qquad \text{(S10)}$$

$$A_{f+1,f} = f\pi^* \qquad 1 \leq f \leq \tau - 1$$

where we index from f = 0 as the first term in the state space, and where π^* is the aggregate cell proliferation rate, which remains constant when HTLV-1 proviral load is at equilibrium. In our system, A does not have any time-dependent factors, and so Eq (S9) has solution

$$\mathbb{P}(X_i;t) = e^{At}\mathbb{P}_{0,i} \qquad \text{(S11)}$$

where $\mathbb{P}_{0,i} = \mathbb{P}(X_i; t = 0)$ is the initial probability distribution and e^{At} is the matrix exponential [9].

Initial Probability Distribution

The initial probability distribution $\mathbb{P}_{0,i} = \mathbb{P}(X_i; t = 0) \in \mathbb{R}^\tau$ [9] can be used to enter a fixed initial value, in which case for initial value $X_i(0) = y_{0,i} \in \{0, \ldots, \tau\}$, $y_i \in \{0, ..., \tau\}$, $\tau \in \mathbb{N}$ we have

$$\mathbb{P}_{0,i} = \delta_{y_i, y_{0,i}} \quad \text{(S12)}$$

where $\delta_{y_i, y_{0,i}}$ [10] is the Kronecker delta such that

$$\delta_{y_i, y_{0,i}} = \begin{cases} 1, & \text{if } y_i = y_{0,i} \\ 0, & \text{if } y_i \neq y_{0,i} \end{cases}.$$

Matrix exponential

Computing the matrix exponential e^{At} is straightforward in principle, but considerably more difficult in practice when the dimension of the matrix is large [11-14]. Compounding this problem, multiple probability distributions at multiple time points are often desired. Fortunately however, the probability distribution for a series of time points can be calculated recursively using a single matrix exponential. More precisely, if the reaction propensities are independent of time, we can compute the matrix exponential e^{As} for a given time step *s*, and then recursively calculate the probability distribution at any time that is a multiple of *s*. Therefore, for $k \in \mathbb{N}_0$, we have the following recurrence relation:

$$\mathbb{P}(X_i; t = s) = e^{As}\mathbb{P}_{0,i}$$

$$\mathbb{P}(X_i; t = ks) = e^{As}\mathbb{P}(X_i; t = (k-1)s)$$

In long form, we have

$$\mathbb{P}(X_i; t = ks) = e^{Aks}\mathbb{P}_{0,i} = \left(e^{As}\right)^k \mathbb{P}_{0,i}$$

This means the computationally expensive [11] matrix exponential e^{As} needs to be calculated once only, and then recursively multiplied *k* times, which incurs significantly less runtime than calculating the matrix exponential *k* times.

It is important to note that there is no source inflow from frequency 0 to frequency 1 in our birth-death process. That is, we do not have a reaction ρ_j such that

$$\rho_j : * \xrightarrow{\lambda} \mathcal{X}$$

for some constant value λ, as a clone cannot proliferate once it has become extinct.

Stochastic threshold frequency F and state space upper limit τ

To allow the possibility that a given clone goes extinct, a stochastic model is necessary; a purely deterministic set of ODEs would not allow HTLV-1 clones to die out. Ideally, each clone would be modelled stochastically, however in practice this is not computationally tractable, and so some clones must be modelled deterministically. The dynamics of large clones can be better approximated than those of small clones by a deterministic process. Further the errors associated with considering a stochastic process deterministically will decrease with the size of the clone. It is also true that larger clones are less likely to die out, and therefore modelling their extinction probability is less important. We took an empirical approach to specifying a threshold frequency F above which clones are modelled deterministically and below which clones are modelled stochastically.

We defined the threshold frequency F in terms of the extinction probability over an arbitrarily long duration. We chose a duration t_{Dur} = 3133 days, as this was the maximum time between a patient's first and last blood samples. Clones with a low probability of extinction over this period can be modelled deterministically without

adversely affecting the modelling of infectious spread. Whereas clones with more than a 1% chance of extinction over this period are modelled stochastically. Therefore *F* is given by

$$F = \min\{ f : \quad \mathbb{P}(X_i(t_{\text{Dur}}) = 0 \mid X_i(0) = f) < 0.01 \quad \} \tag{S13}$$

given the rates of cell death and mitotic spread, and density dependency (S3A Fig). The upper limit *τ* of clone frequency is chosen to be sufficiently large not to constrain the trajectory of a growing clone. If this upper limit is too small, then the growth of a clone may be artificially limited, because it may "bounce" off the upper limit, as this cannot be exceeded by a stochastically modelled clone (Fig 3B). S3B Fig shows that a clone given a starting frequency *F* = 460 has a negligible probability of reaching frequency *τ* = 1500 after 3133 days, and therefore we chose *τ* = 1500 as the upper limit on the clone state space.

Hybrid model propagation: Strang splitting

For clones above threshold frequency *F*, we assume the expected values from the deterministic ODEs provide an adequate description of their behaviour. We thus partition our system of the within-host dynamics of HTLV-1 infection into two constituent systems: a deterministic system *D(t)* modelled by a system of ODEs and a stochastic system *σ(t)* modelled by multiple master equations. We propagate these systems concurrently as a hybrid model, using the following procedure of "Strang splitting" [15] for a given time step t_n of duration *h* (formulation below taken from [1]):

1. Half time step in deterministic *D(t)*: Solve deterministic process in time interval $[t_n, t_n + h/2]$; keep *σ(t)* constant.

2. Full time step in stochastic $\sigma(t)$: Solve stochastic process (i.e. clone-specific master equations) in time interval $[t_n, t_{n+1}]$ and keep $D(t_n + h/2)$ constant.
3. Next half time step in deterministic system $D(t)$: Solve deterministic process in time interval $[t_n + h/2, t_{n+1}]$ and keep $\sigma(t)$ constant.

The error associated in not propagating both systems exactly simultaneously is a function of the length of the time step *h* [1]. Modelling the whole system for a single time step consists of steps 1 to 3 (Fig 2B).

REFERENCES

1. Jahnke T, Kreim M. Error bound for piecewise deterministic processes modeling stochastic reaction systems. Multiscale Modeling & Simulation. 2012;10(4):1119-47.

2. Jahnke T, Sunkara V. Error Bound for Hybrid Models of Two-Scaled Stochastic Reaction Systems. In: Dahlke S, Dahmen W, Griebel M, Hackbusch W, Ritter K, Schneider R, et al., editors. Extraction of Quantifiable Information from Complex Systems. Cham: Springer International Publishing; 2014. p. 303-19.

3. Gillespie DT. Exact stochastic simulation of coupled chemical reactions. The Journal of Physical Chemistry. 1977;81(25):2340-61. doi: 10.1021/j100540a008.

4. Van Kampen NG. Stochastic processes in physics and chemistry: Elsevier; 1992.

5. Gillespie DT. A rigorous derivation of the chemical master equation. Physica A: Statistical Mechanics and its Applications. 1992;188(1):404-25.

6. Jahnke T. On reduced models for the chemical master equation. Multiscale Modeling & Simulation. 2011;9(4):1646-76.

7. Jahnke T, Huisinga W. A dynamical low-rank approach to the chemical master equation. Bulletin of mathematical biology. 2008;70(8):2283-302.

8. Hegland M, Burden C, Santoso L, MacNamara S, Booth H. A solver for the stochastic master equation applied to gene regulatory networks. Journal of computational and applied mathematics. 2007;205(2):708-24.

9. Stewart WJ. Introduction to the numerical solutions of Markov chains: Princeton Univ. Press; 1994.

10. Peleš S, Munsky B, Khammash M. Reduction and solution of the chemical master equation using time scale separation and finite state projection. The Journal of chemical physics. 2006;125(20):204104.

11. Moler C, Van Loan C. Nineteen dubious ways to compute the exponential of a matrix. SIAM review. 1978;20(4):801-36.

12. Moler C, Van Loan C. Nineteen dubious ways to compute the exponential of a matrix, twenty-five years later. SIAM review. 2003;45(1):3-49.

13. Higham NJ. The scaling and squaring method for the matrix exponential revisited. SIAM review. 2009;51(4):747-64.

14. Lopez L, Simoncini V. Analysis of projection methods for rational function approximation to the matrix exponential. SIAM Journal on Numerical Analysis. 2006;44(2):613-35.

15. Strang G. On the construction and comparison of difference schemes. SIAM Journal on Numerical Analysis. 1968;5(3):506-17.
