# Supplementary material for: The relative contributions of infectious and mitotic spread to HTLV-1 persistence
Source: PLoS Comput Biol. 2020 Sep 17;16(9):e1007470. doi: 10.1371/journal.pcbi.1007470 (PMC7524007; doi:10.1371/journal.pcbi.1007470)
Supplement: S3 Text — (PDF) [file pcbi.1007470.s003.pdf]

Sensitivity of hybrid model to length of time step

To ensure the Strang splitting error is sufficiently small, we ran the model for multiple sets of parameters (representing "growth", "equilibrium" and "death") and multiple time step lengths (from 10 days to one day, and also 0.5, 0.25, and 0.1 days). In each instance, we recorded a number of observables, and chose h such that decreasing the time step further did not substantially affect these observables. We chose the observables to be the number of clones at a given duration of the hybrid (500 days); the number of infected cells at this duration; and the minimum number of clones over the model run. S4 Fig plots each observable as a function of the inverse of h. For all parameter sets, each observable is flat when plotted against time step. We conclude that a time step of h = 1 day results in a sufficiently small splitting error.

Comparison of hybrid and purely stochastic model

It is not possible to model the entire system HTLV-1 T cell clones stochastically, due to the prohibitively high frequencies of large clones. However, we consider a greatly reduced system for which a purely stochastic model and a hybrid stochastic and deterministic model can be compared.

The reduced system omits all clones of frequency greater than 460 cells, which substantially reduces the number of infected cells, but reduces the number of clones less drastically. When considered as a hybrid, clones above F = 100 are considered deterministically. We fit the purely stochastic simulation and the hybrid as before for example patients for t_{Dur} = 100 days.

We find that that the purely stochastic and the hybrid models give very similar values of both the values of r_I and expected number of clones over time (S5 Fig), for each example patient. It must be stressed that in these simulations, the fitted value of r_I is much higher than for the actual system of HTLV-1 clones, because the number of infected cells in the reduced system is approximately 5 orders of magnitude lower, and new clones are modelled as a mass-action process.

We would expect the errors associated with using a hybrid instead of a purely stochastic model to be lower for the actual system of HTLV-1 clones because there clones and cells are much larger and less susceptible by random variation. We conclude that the error associated with using a hybrid model is acceptable.
