# Supplementary material for: The relative contributions of infectious and mitotic spread to HTLV-1 persistence
Source: PLoS Comput Biol. 2020 Sep 17;16(9):e1007470. doi: 10.1371/journal.pcbi.1007470 (PMC7524007; doi:10.1371/journal.pcbi.1007470)
Supplement: S1 Table — (PDF) [file pcbi.1007470.s004.pdf]

S1 Table. Patient sample characteristics and diversity estimates.

Patient (Disease Status‡)	Blood sample [Date (replicate number)]	Proviral load (no. HTLV-1+ cells per 10,000 PBMCs) [1]	Number of clones [Observed [1]/ Estimated [2]]	Estimated percentage of clones that are singletons (clones with one infected cell only [2]
**1 (AC)**	07/12/2000 (1)	400	993 / 55898	9.6
**1 (AC)**	07/12/2000 (2)	400	1047 / 62527	9.7
**1 (AC)**	07/12/2000 (3)	400	935 / 53598	9.5
**1 (AC)**	13/12/2004 (1)	490	1187 / 54610	9.2
**1 (AC)**	13/12/2004 (2)	490	1423 / 56531	9.1
**1 (AC)**	13/12/2004 (3)	490	1047 / 37353	8.3
**1 (AC)**	17/11/2008 (1)	360	1273 / 37295	8.3
**1 (AC)**	17/11/2008 (2)	360	896 / 56753	9.8
**1 (AC)**	17/11/2008 (3)	360	1122 / 41432	8.8
**2 (UV)**	03/04/2000 (1)	130	698 / 19773	7.9
**2 (UV)**	03/04/2000 (2)	130	622 / 16882	7.7
**2 (UV)**	03/04/2000 (3)	130	559 / 21705	8.5
**2 (UV)**	16/06/2005 (1)	120	550 / 23319	8.6
**2 (UV)**	16/06/2005 (2)	120	778 / 18211	7.7
**2 (UV)**	16/06/2005 (3)	120	638 / 23869	8.6
**2 (UV)**	22/09/2008 (1)	150	590 / 11090	6.6
**2 (UV)**	22/09/2008 (2)	150	523 / 18275	8.3
**2 (UV)**	22/09/2008 (3)	150	541 / 18102	8.2
**3 (HAM)**	24/09/2001 (1)	350	1226 / 58084	9.3
**3 (HAM)**	24/09/2001 (2)	350	1156 / 78040	9.9
**3 (HAM)**	24/09/2001 (3)	350	1391 / 71505	9.6
**3 (HAM)**	04/01/2007 (1)	390	1464 / 70338	9.5
**3 (HAM)**	04/01/2007 (2)	390	1555 / 61548	9.2
**3 (HAM)**	04/01/2007 (3)	390	1085 / 56442	9.4
**3 (HAM)**	14/04/2008 (1)	220	875 / 43305	9.4
**3 (HAM)**	14/04/2008 (2)	220	974 / 53960	9.7
**3 (HAM)**	14/04/2008 (3)	220	922 / 45946	9.5
**4 (HAM)**	09/11/2000 (1)	930	1562 / 36965	7.3
**4 (HAM)**	09/11/2000 (2)	930	1576 / 34842	7.2
**4 (HAM)**	09/11/2000 (3)	930	1574 / 30292	6.8
**4 (HAM)**	16/03/2005 (1)	960	1833 / 37273	7.2
**4 (HAM)**	16/03/2005 (2)	960	2406 / 33819	6.6
**4 (HAM)**	16/03/2005 (3)	960	1918 / 44637	7.6
**4 (HAM)**	02/10/2008 (1)	870	1862 / 40070	7.4
**4 (HAM)**	02/10/2008 (2)	870	1788 / 29684	6.7
**4 (HAM)**	02/10/2008 (3)	870	1754 / 43982	7.6
**5 (HAM)**	12/06/2000 (1)	160	568 / 17656	8.1
**5 (HAM)**	12/06/2000 (2)	160	647 / 21418	8.2
**5 (HAM)**	12/06/2000 (3)	160	628 / 19158	8
**5 (HAM)**	01/12/2005 (1)	180	569 / 13454	7.2

**5 (HAM)**	01/12/2005 (2)	180	726 / 15333	7.5
**5 (HAM)**	01/12/2005 (3)	180	580 / 13744	7.5
**5 (HAM)**	03/11/2008 (1)	140	564 / 17674	8.4
**5 (HAM)**	03/11/2008 (2)	140	545 / 16081	8.1
**5 (HAM)**	03/11/2008 (3)	140	551 / 13849	7.7
**6 (HAM)**	19/03/2001 (1)	180	830 / 21720	7.9
**6 (HAM)**	19/03/2001 (2)	180	691 / 16542	7.4
**6 (HAM)**	19/03/2001 (3)	180	802 / 20321	7.7
**6 (HAM)**	23/09/2004 (1)	240	837 / 12750	6.4
**6 (HAM)**	23/09/2004 (2)	240	832 / 19776	7.5
**6 (HAM)**	23/09/2004 (3)	240	698 / 15481	7
**6 (HAM)**	18/06/2007 (1)	140	581 / 9810	6.4
**6 (HAM)**	18/06/2007 (2)	140	591 / 17631	8
**6 (HAM)**	18/06/2007 (3)	140	639 / 9121	6.1
**7 (HAM)**	15/01/2001 (1)	2210	3677 / 163991	8.9
**7 (HAM)**	15/01/2001 (2)	2210	3251 / 163857	9
**7 (HAM)**	15/01/2001 (3)	2210	3687 / 172011	9
**7 (HAM)**	03/02/2005 (1)	2020	3714 / 146340	8.6
**7 (HAM)**	03/02/2005 (2)	2020	5482 / 131888	7.9
**7 (HAM)**	03/02/2005 (3)	2020	2981 / 157621	8.9
**7 (HAM)**	04/12/2008 (1)	2000	4703 / 136632	8.2
**7 (HAM)**	04/12/2008 (2)	2000	3992 / 131033	8.2
**7 (HAM)**	04/12/2008 (3)	2000	3359 / 166248	9
**8 (HAM)**	25/05/2000 (1)	2300	2089 / 67798	8
**8 (HAM)**	25/05/2000 (2)	2300	2443 / 56116	7.3
**8 (HAM)**	25/05/2000 (3)	2300	2916 / 53761	6.9
**8 (HAM)**	04/09/2003 (1)	1500	2641 / 50085	7.2
**8 (HAM)**	04/09/2003 (2)	1500	3235 / 39501	6.3
**8 (HAM)**	04/09/2003 (3)	1500	3502 / 38443	6.1
**8 (HAM)**	22/12/2008 (1)	1460	2282 / 52252	7.6
**8 (HAM)**	22/12/2008 (2)	1460	2004 / 62329	8.2
**8 (HAM)**	22/12/2008 (3)	1460	2129 / 49931	7.5
**9 (HAM)**	06/11/2000 (1)	1990	1154 / 138062	10
**9 (HAM)**	06/11/2000 (2)	1990	1946 / 131701	9.5
**9 (HAM)**	06/11/2000 (3)	1990	1923 / 123403	9.5
**9 (HAM)**	01/07/2004 (1)	1410	1666 / 165332	10
**9 (HAM)**	01/07/2004 (2)	1410	2732 / 105526	8.9
**9 (HAM)**	01/07/2004 (3)	1410	1865 / 160477	10
**9 (HAM)**	15/03/2007 (1)	2080	1294 / 186468	12
**9 (HAM)**	15/03/2007 (2)	2080	1169 / 130672	11
**9 (HAM)**	15/03/2007 (3)	2080	1241 / 136643	11
**10 (HAM)**	05/04/2004 (1)	940	1598 / 70437	8.9
**10 (HAM)**	05/04/2004 (2)	940	1651 / 84639	9.2
**10 (HAM)**	05/04/2004 (3)	940	1609 / 83176	9.3
**10 (HAM)**	13/02/2007 (1)	770	2223 / 61504	8.3
**10 (HAM)**	13/02/2007 (2)	770	2409 / 63395	8.3

**10**	**(HAM)**	13/02/2007 (3)	770	2008 / 67936	8.7
**10**	**(HAM)**	18/11/2008 (1)	730	1412 / 64961	9
**10**	**(HAM)**	18/11/2008 (2)	730	1941 / 67440	8.7
**10**	**(HAM)**	18/11/2008 (3)	730	1662 / 56583	8.5
**11**	**(HAM)**	06/03/2000 (1)	430	1238 / 45190	8.4
**11**	**(HAM)**	06/03/2000 (2)	430	1371 / 66409	9.1
**11**	**(HAM)**	06/03/2000 (3)	430	1727 / 79065	9.3
**11**	**(HAM)**	15/05/2003 (1)	750	1805 / 53637	8
**11**	**(HAM)**	15/05/2003 (2)	750	2548 / 48187	7.3
**11**	**(HAM)**	15/05/2003 (3)	750	1771 / 70610	8.8
**11**	**(HAM)**	29/09/2008 (1)	890	2103 / 52457	7.7
**11**	**(HAM)**	29/09/2008 (2)	890	2155 / 64498	8.2
**11**	**(HAM)**	29/09/2008 (3)	890	2204 / 52248	7.6

‡ Disease status: AC = asymptomatic carrier. UV = uveitis (non-HAM/TSP); HAM = HAM/TSP

REFERENCES

1. Gillet NA, Malani N, Melamed A, Gormley N, Carter R, Bentley D, et al. The host genomic environment of the provirus determines the abundance of HTLV-1-infected T-cell clones. Blood. 2011;117(11):3113-22. Epub 2011/01/14. doi: https://doi.org/10.1182/blood-2010-10-312926. PubMed PMID: 21228324; PubMed Central PMCID: PMC3062313.

2. Laydon DJ, Melamed A, Sim A, Gillet NA, Sim K, Darko S, et al. Quantification of HTLV-1 Clonality and TCR Diversity. PLoS Comput Biol. 2014;10(6):e1003646. doi: 10.1371/journal.pcbi.1003646.
