# Supplementary material for: The relative contributions of infectious and mitotic spread to HTLV-1 persistence
Source: PLoS Comput Biol. 2020 Sep 17;16(9):e1007470. doi: 10.1371/journal.pcbi.1007470 (PMC7524007; doi:10.1371/journal.pcbi.1007470)
Supplement: S2 Table — (PDF) [file pcbi.1007470.s005.pdf]

S2 Table. Notation used in models of HTLV-1 within host persistence.

Notation	Description
**General**	
i	clone index
$S(t)$	Expected number of HTLV-1 clones at time t
$N(t)$	Expected number of HTLV-1 infected cells at time t
π	Per-capita rate of mitotic spread (infected cell proliferation)
δ	Per-capita rate of infected cell death
r_I	Per-capita rate of infectious spread (*de novo* infection)
R	Ratio of infectious to mitotic spread
f	Clone frequency (index)
n_f	Number of clones of frequency f
**Method 1: Full Simulation Hybrid Model**	
$x_i(t)$	Expected number of infected cells in clone i at time t
K	Density dependency parameter. Infected cell proliferation rates are half maximal when number of infected cells $N(t) = K$
$\pi^*(t)$	Density-dependent proliferation rate
c, C	Index and number of possible reactions of system respectively
ρ_c	Mapping of reaction c
v_c	Stoichiometric vector of reaction c
α_c	Propensity function of reaction c
S_{max}	Integer chosen to always be larger than $S(t)$, for all t
$X(t)$	Random variable denoting the state of the entire system (all clones and their frequencies) at time t
$X_i(t)$	Random variable denoting the frequency of clone i at time t
y	Potential state of random variable $X(t)$
y_0	Initial state of all clones and their frequencies
τ	Maximum frequency that stochastically modelled clone allowed to take

F	Threshold frequency above which clones are modelled deterministically and below which clones are modelled stochastically
$b(t)$	Number of clone births at time t
$E(t)$	Expected number of clones deaths/extinctions at time t
S_0	Number of clones at time zero
h	Length of time step in hybrid model
$D(t)$	State of deterministic part of hybrid model at time t
$\sigma(t)$	State of stochastic part of hybrid model at time t

Method 2: Upper Bound Approximation

δ_S	Clone death rate
$\hat{\delta}_{small}$, $\hat{\delta}_{large}$	Aggregate clone death rate of small and large clones respectively
$r_{I,Supremum}$	Estimated supremum of infectious spread rate, assuming cell death rate δ applies to all clones, irrespective of size
$R_{Supremum}$	Estimated ratio of infectious spread to mitotic spread, assuming cell death rate δ applies to all clones, irrespective of size
f_{max}	Frequency above which clones do not contribute to clone death rate
$r_{I,\ fmax}$	Estimated upper bound of infectious spread rate, assuming clones above frequency f_{max} do not contribute to clone death rate
R_{fmax}	Estimated ratio of infectious spread to mitotic spread, assuming clones above frequency f_{max} do not contribute to clone death rate

Method 3: Occupancy class model

M	Rate of mitotic events in deterministic clone class model
I	Rate of infectious events in deterministic clone class model
C_f	Number of infected cells in class size n_f in deterministic clone class model ($C_f = fn_f$)
R_1	Ratio of infectious to mitotic spread in terms of proportion of cells that are singletons
R_2	Ratio of infectious to mitotic spread in terms of species richness
