# Supplementary material for: The relative contributions of infectious and mitotic spread to HTLV-1 persistence
Source: PLoS Comput Biol. 2020 Sep 17;16(9):e1007470. doi: 10.1371/journal.pcbi.1007470 (PMC7524007; doi:10.1371/journal.pcbi.1007470)
Supplement: S3 Table — (PDF) [file pcbi.1007470.s006.pdf]

S3 Table. Hybrid model sensitivity to proliferation and death rates.

Patient (Disease Status‡)	Blood sample [Date (replicate number)]	Infectious spread rate r_I (d^{-1})			Ratio of infectious spread to mitotic spread		
		Central ($\pi^* = \delta =$ 0.0316)	Lower ($\pi^* = \delta =$ 0.0158)	Higher ($\pi^* = \delta =$ 0.0632)	Central ($\pi^* = \delta =$ 0.0316)	Lower ($\pi^* = \delta =$ 0.0158)	Higher ($\pi^* = \delta =$ 0.0632)
1 (AC)	07/12/2000 (1)	1.20E-09	6.14E-10	2.35E-09	3.81E-08	3.89E-08	3.71E-08
1 (AC)	07/12/2000 (2)	1.36E-09	6.96E-10	2.65E-09	4.32E-08	4.41E-08	4.20E-08
1 (AC)	07/12/2000 (3)	1.15E-09	5.89E-10	2.25E-09	3.65E-08	3.73E-08	3.56E-08
1 (AC)	13/12/2004 (1)	9.31E-10	4.74E-10	1.81E-09	2.95E-08	3.00E-08	2.87E-08
1 (AC)	13/12/2004 (2)	9.58E-10	4.87E-10	1.87E-09	3.03E-08	3.08E-08	2.96E-08
1 (AC)	13/12/2004 (3)	5.92E-10	3.00E-10	1.16E-09	1.87E-08	1.90E-08	1.83E-08
1 (AC)	17/11/2008 (1)	8.03E-10	4.07E-10	1.57E-09	2.54E-08	2.58E-08	2.48E-08
1 (AC)	17/11/2008 (2)	1.39E-09	7.08E-10	2.70E-09	4.39E-08	4.48E-08	4.27E-08
1 (AC)	17/11/2008 (3)	9.31E-10	4.73E-10	1.82E-09	2.95E-08	3.00E-08	2.88E-08
2 (UV)	03/04/2000 (1)	1.14E-09	5.77E-10	2.23E-09	3.60E-08	3.65E-08	3.53E-08
2 (UV)	03/04/2000 (2)	9.46E-10	4.78E-10	1.85E-09	2.99E-08	3.03E-08	2.93E-08
2 (UV)	03/04/2000 (3)	1.32E-09	6.68E-10	2.57E-09	4.16E-08	4.23E-08	4.07E-08
2 (UV)	16/06/2005 (1)	1.56E-09	7.90E-10	3.04E-09	4.92E-08	5.00E-08	4.81E-08
2 (UV)	16/06/2005 (2)	1.11E-09	5.61E-10	2.18E-09	3.51E-08	3.55E-08	3.44E-08
2 (UV)	16/06/2005 (3)	1.59E-09	8.05E-10	3.10E-09	5.02E-08	5.10E-08	4.90E-08
2 (UV)	22/09/2008 (1)	4.77E-10	2.40E-10	9.41E-10	1.51E-08	1.52E-08	1.49E-08
2 (UV)	22/09/2008 (2)	9.47E-10	4.80E-10	1.85E-09	3.00E-08	3.04E-08	2.93E-08
2 (UV)	22/09/2008 (3)	9.30E-10	4.71E-10	1.82E-09	2.94E-08	2.98E-08	2.88E-08
3 (HAM)	24/09/2001 (1)	1.40E-09	7.16E-10	2.74E-09	4.45E-08	4.53E-08	4.33E-08
3 (HAM)	24/09/2001 (2)	1.98E-09	1.01E-09	3.85E-09	6.27E-08	6.40E-08	6.09E-08
3 (HAM)	24/09/2001 (3)	1.77E-09	9.00E-10	3.44E-09	5.59E-08	5.70E-08	5.44E-08
3 (HAM)	04/01/2007 (1)	1.55E-09	7.89E-10	3.01E-09	4.90E-08	4.99E-08	4.77E-08
3 (HAM)	04/01/2007 (2)	1.32E-09	6.73E-10	2.58E-09	4.18E-08	4.26E-08	4.08E-08
3 (HAM)	04/01/2007 (3)	1.24E-09	6.30E-10	2.41E-09	3.91E-08	3.99E-08	3.81E-08
3 (HAM)	14/04/2008 (1)	1.68E-09	8.58E-10	3.28E-09	5.33E-08	5.43E-08	5.19E-08
3 (HAM)	14/04/2008 (2)	2.14E-09	1.09E-09	4.17E-09	6.78E-08	6.92E-08	6.60E-08
3 (HAM)	14/04/2008 (3)	1.79E-09	9.11E-10	3.48E-09	5.66E-08	5.77E-08	5.51E-08
4 (HAM)	09/11/2000 (1)	2.77E-10	1.40E-10	5.44E-10	8.77E-09	8.85E-09	8.61E-09
4 (HAM)	09/11/2000 (2)	2.60E-10	1.31E-10	5.11E-10	8.23E-09	8.31E-09	8.08E-09
4 (HAM)	09/11/2000 (3)	2.14E-10	1.08E-10	4.22E-10	6.78E-09	6.84E-09	6.67E-09
4 (HAM)	16/03/2005 (1)	2.68E-10	1.35E-10	5.26E-10	8.47E-09	8.55E-09	8.32E-09
4 (HAM)	16/03/2005 (2)	2.27E-10	1.14E-10	4.48E-10	7.19E-09	7.25E-09	7.08E-09
4 (HAM)	16/03/2005 (3)	3.35E-10	1.70E-10	6.57E-10	1.06E-08	1.07E-08	1.04E-08
4 (HAM)	02/10/2008 (1)	3.26E-10	1.65E-10	6.41E-10	1.03E-08	1.04E-08	1.01E-08
4 (HAM)	02/10/2008 (2)	2.22E-10	1.12E-10	4.38E-10	7.03E-09	7.08E-09	6.93E-09
4 (HAM)	02/10/2008 (3)	3.67E-10	1.86E-10	7.20E-10	1.16E-08	1.18E-08	1.14E-08
5 (HAM)	12/06/2000 (1)	8.35E-10	4.23E-10	1.64E-09	2.64E-08	2.68E-08	2.59E-08
5 (HAM)	12/06/2000 (2)	1.03E-09	5.22E-10	2.02E-09	3.26E-08	3.31E-08	3.19E-08

5 (HAM)	12/06/2000 (3)	9.00E-10	4.56E-10	1.76E-09	2.85E-08	2.89E-08	2.79E-08
5 (HAM)	01/12/2005 (1)	5.20E-10	2.63E-10	1.02E-09	1.65E-08	1.66E-08	1.62E-08
5 (HAM)	01/12/2005 (2)	6.08E-10	3.07E-10	1.19E-09	1.92E-08	1.94E-08	1.89E-08
5 (HAM)	01/12/2005 (3)	5.42E-10	2.74E-10	1.06E-09	1.72E-08	1.74E-08	1.68E-08
5 (HAM)	03/11/2008 (1)	9.91E-10	5.03E-10	1.94E-09	3.14E-08	3.18E-08	3.06E-08
5 (HAM)	03/11/2008 (2)	8.73E-10	4.42E-10	1.71E-09	2.76E-08	2.80E-08	2.71E-08
5 (HAM)	03/11/2008 (3)	7.23E-10	3.66E-10	1.42E-09	2.29E-08	2.32E-08	2.24E-08
6 (HAM)	19/03/2001 (1)	8.98E-10	4.55E-10	1.76E-09	2.84E-08	2.88E-08	2.79E-08
6 (HAM)	19/03/2001 (2)	6.53E-10	3.30E-10	1.28E-09	2.07E-08	2.09E-08	2.03E-08
6 (HAM)	19/03/2001 (3)	8.24E-10	4.17E-10	1.62E-09	2.61E-08	2.64E-08	2.56E-08
6 (HAM)	23/09/2004 (1)	3.35E-10	1.69E-10	6.61E-10	1.06E-08	1.07E-08	1.05E-08
6 (HAM)	23/09/2004 (2)	5.89E-10	2.98E-10	1.16E-09	1.86E-08	1.88E-08	1.83E-08
6 (HAM)	23/09/2004 (3)	4.37E-10	2.21E-10	8.60E-10	1.38E-08	1.40E-08	1.36E-08
6 (HAM)	18/06/2007 (1)	4.41E-10	2.22E-10	8.70E-10	1.40E-08	1.40E-08	1.38E-08
6 (HAM)	18/06/2007 (2)	9.50E-10	4.82E-10	1.86E-09	3.01E-08	3.05E-08	2.94E-08
6 (HAM)	18/06/2007 (3)	3.94E-10	1.98E-10	7.78E-10	1.25E-08	1.25E-08	1.23E-08
7 (HAM)	15/01/2001 (1)	6.06E-10	3.08E-10	1.18E-09	1.92E-08	1.95E-08	1.87E-08
7 (HAM)	15/01/2001 (2)	6.09E-10	3.10E-10	1.19E-09	1.93E-08	1.96E-08	1.88E-08
7 (HAM)	15/01/2001 (3)	6.40E-10	3.25E-10	1.25E-09	2.02E-08	2.06E-08	1.97E-08
7 (HAM)	03/02/2005 (1)	5.74E-10	2.92E-10	1.12E-09	1.82E-08	1.85E-08	1.77E-08
7 (HAM)	03/02/2005 (2)	4.88E-10	2.47E-10	9.56E-10	1.55E-08	1.57E-08	1.51E-08
7 (HAM)	03/02/2005 (3)	6.39E-10	3.25E-10	1.25E-09	2.02E-08	2.06E-08	1.97E-08
7 (HAM)	04/12/2008 (1)	5.26E-10	2.67E-10	1.03E-09	1.67E-08	1.69E-08	1.63E-08
7 (HAM)	04/12/2008 (2)	5.04E-10	2.56E-10	9.87E-10	1.60E-08	1.62E-08	1.56E-08
7 (HAM)	04/12/2008 (3)	6.85E-10	3.48E-10	1.34E-09	2.17E-08	2.20E-08	2.11E-08
8 (HAM)	25/05/2000 (1)	2.21E-10	1.12E-10	4.32E-10	6.98E-09	7.08E-09	6.83E-09
8 (HAM)	25/05/2000 (2)	1.70E-10	8.61E-11	3.35E-10	5.39E-09	5.45E-09	5.29E-09
8 (HAM)	25/05/2000 (3)	1.55E-10	7.84E-11	3.06E-10	4.92E-09	4.96E-09	4.84E-09
8 (HAM)	04/09/2003 (1)	2.31E-10	1.17E-10	4.54E-10	7.30E-09	7.38E-09	7.18E-09
8 (HAM)	04/09/2003 (2)	1.63E-10	8.20E-11	3.21E-10	5.16E-09	5.19E-09	5.09E-09
8 (HAM)	04/09/2003 (3)	1.55E-10	7.79E-11	3.07E-10	4.91E-09	4.93E-09	4.86E-09
8 (HAM)	22/12/2008 (1)	2.59E-10	1.31E-10	5.07E-10	8.19E-09	8.29E-09	8.02E-09
8 (HAM)	22/12/2008 (2)	3.28E-10	1.66E-10	6.43E-10	1.04E-08	1.05E-08	1.02E-08
8 (HAM)	22/12/2008 (3)	2.46E-10	1.24E-10	4.82E-10	7.77E-09	7.86E-09	7.62E-09
9 (HAM)	06/11/2000 (1)	6.34E-10	3.24E-10	1.23E-09	2.00E-08	2.05E-08	1.94E-08
9 (HAM)	06/11/2000 (2)	5.68E-10	2.90E-10	1.11E-09	1.80E-08	1.83E-08	1.75E-08
9 (HAM)	06/11/2000 (3)	5.30E-10	2.70E-10	1.03E-09	1.68E-08	1.71E-08	1.63E-08
9 (HAM)	01/07/2004 (1)	1.08E-09	5.54E-10	2.10E-09	3.42E-08	3.50E-08	3.32E-08
9 (HAM)	01/07/2004 (2)	6.13E-10	3.12E-10	1.20E-09	1.94E-08	1.97E-08	1.89E-08
9 (HAM)	01/07/2004 (3)	1.03E-09	5.29E-10	2.01E-09	3.28E-08	3.35E-08	3.18E-08
9 (HAM)	15/03/2007 (1)	9.19E-10	4.74E-10	1.77E-09	2.91E-08	3.00E-08	2.80E-08
9 (HAM)	15/03/2007 (2)	5.86E-10	3.00E-10	1.13E-09	1.85E-08	1.90E-08	1.80E-08
9 (HAM)	15/03/2007 (3)	6.15E-10	3.15E-10	1.19E-09	1.95E-08	1.99E-08	1.89E-08
10 (HAM)	05/04/2004 (1)	6.10E-10	3.10E-10	1.19E-09	1.93E-08	1.96E-08	1.88E-08
10 (HAM)	05/04/2004 (2)	7.56E-10	3.85E-10	1.47E-09	2.39E-08	2.44E-08	2.33E-08

10 (HAM)	05/04/2004 (3)	7.45E-10	3.79E-10	1.45E-09	2.36E-08	2.40E-08	2.30E-08
10 (HAM)	13/02/2007 (1)	6.19E-10	3.14E-10	1.21E-09	1.96E-08	1.99E-08	1.91E-08
10 (HAM)	13/02/2007 (2)	6.36E-10	3.22E-10	1.24E-09	2.01E-08	2.04E-08	1.97E-08
10 (HAM)	13/02/2007 (3)	7.08E-10	3.60E-10	1.38E-09	2.24E-08	2.28E-08	2.19E-08
10 (HAM)	18/11/2008 (1)	7.34E-10	3.74E-10	1.43E-09	2.32E-08	2.37E-08	2.27E-08
10 (HAM)	18/11/2008 (2)	7.42E-10	3.77E-10	1.45E-09	2.35E-08	2.38E-08	2.29E-08
10 (HAM)	18/11/2008 (3)	6.11E-10	3.10E-10	1.19E-09	1.93E-08	1.96E-08	1.89E-08
11 (HAM)	06/03/2000 (1)	8.20E-10	4.16E-10	1.60E-09	2.60E-08	2.63E-08	2.54E-08
11 (HAM)	06/03/2000 (2)	1.29E-09	6.56E-10	2.51E-09	4.08E-08	4.15E-08	3.98E-08
11 (HAM)	06/03/2000 (3)	1.55E-09	7.90E-10	3.02E-09	4.91E-08	5.00E-08	4.78E-08
11 (HAM)	15/05/2003 (1)	5.36E-10	2.71E-10	1.05E-09	1.70E-08	1.72E-08	1.66E-08
11 (HAM)	15/05/2003 (2)	4.50E-10	2.27E-10	8.83E-10	1.42E-08	1.44E-08	1.40E-08
11 (HAM)	15/05/2003 (3)	7.60E-10	3.86E-10	1.48E-09	2.40E-08	2.44E-08	2.35E-08
11 (HAM)	29/09/2008 (1)	4.29E-10	2.17E-10	8.42E-10	1.36E-08	1.37E-08	1.33E-08
11 (HAM)	29/09/2008 (2)	5.58E-10	2.83E-10	1.09E-09	1.76E-08	1.79E-08	1.73E-08
11 (HAM)	29/09/2008 (3)	4.24E-10	2.14E-10	8.31E-10	1.34E-08	1.36E-08	1.32E-08

‡ Disease status: AC = asymptomatic carrier. UV = uveitis (non-HAM/TSP); HAM = HAM/TSP
