# Supplementary figures and images for: The relative contributions of infectious and mitotic spread to HTLV-1 persistence
Source: PLoS Comput Biol. 2020 Sep 17;16(9):e1007470. doi: 10.1371/journal.pcbi.1007470 (PMC7524007; doi:10.1371/journal.pcbi.1007470)

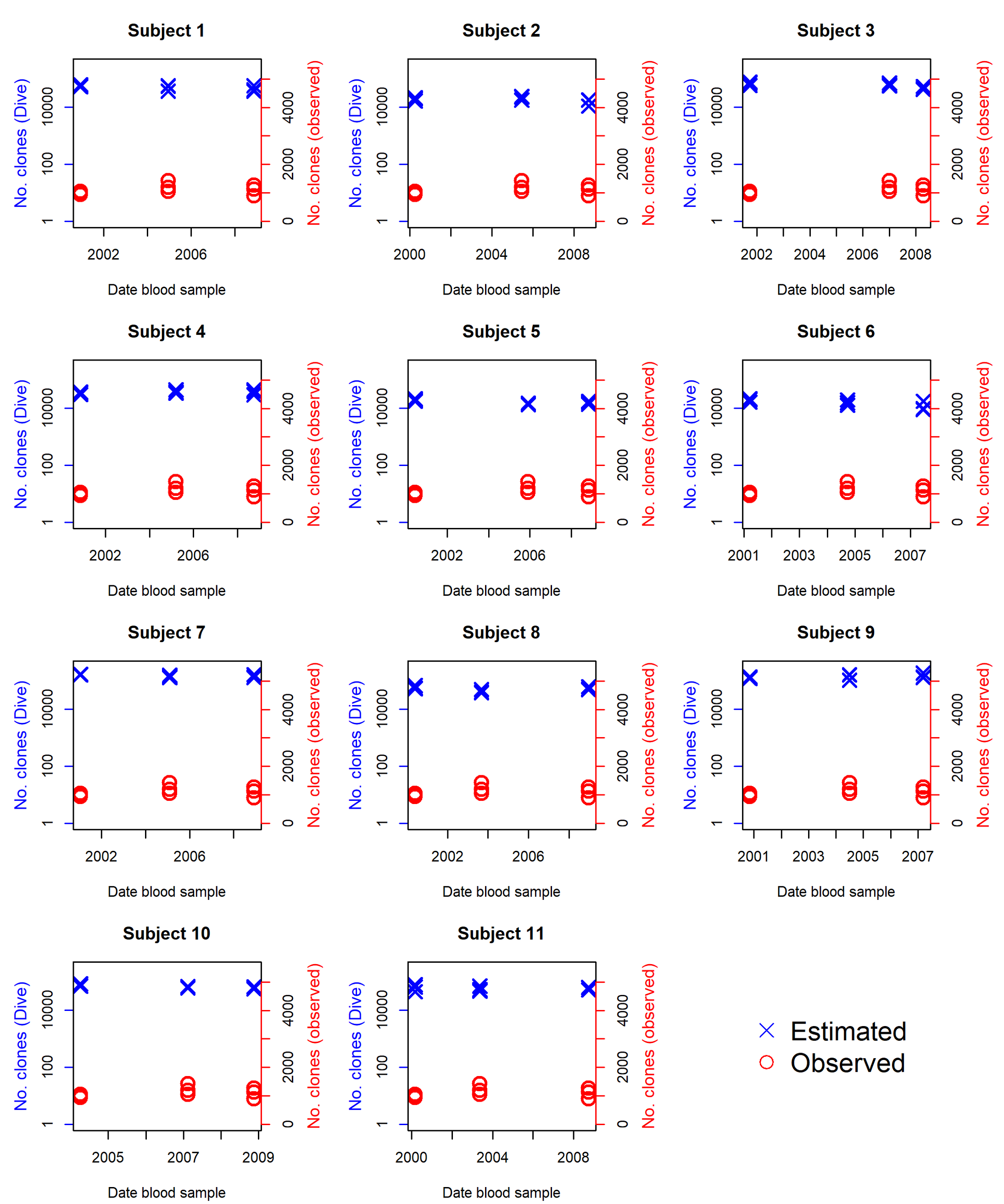

Supplement: S1 Fig — Estimated diversity is shown in blue (left hand y-axes) and observed diversity is shown in red (right-hand y-axes). (TIF) [file pcbi.1007470.s007.tif]

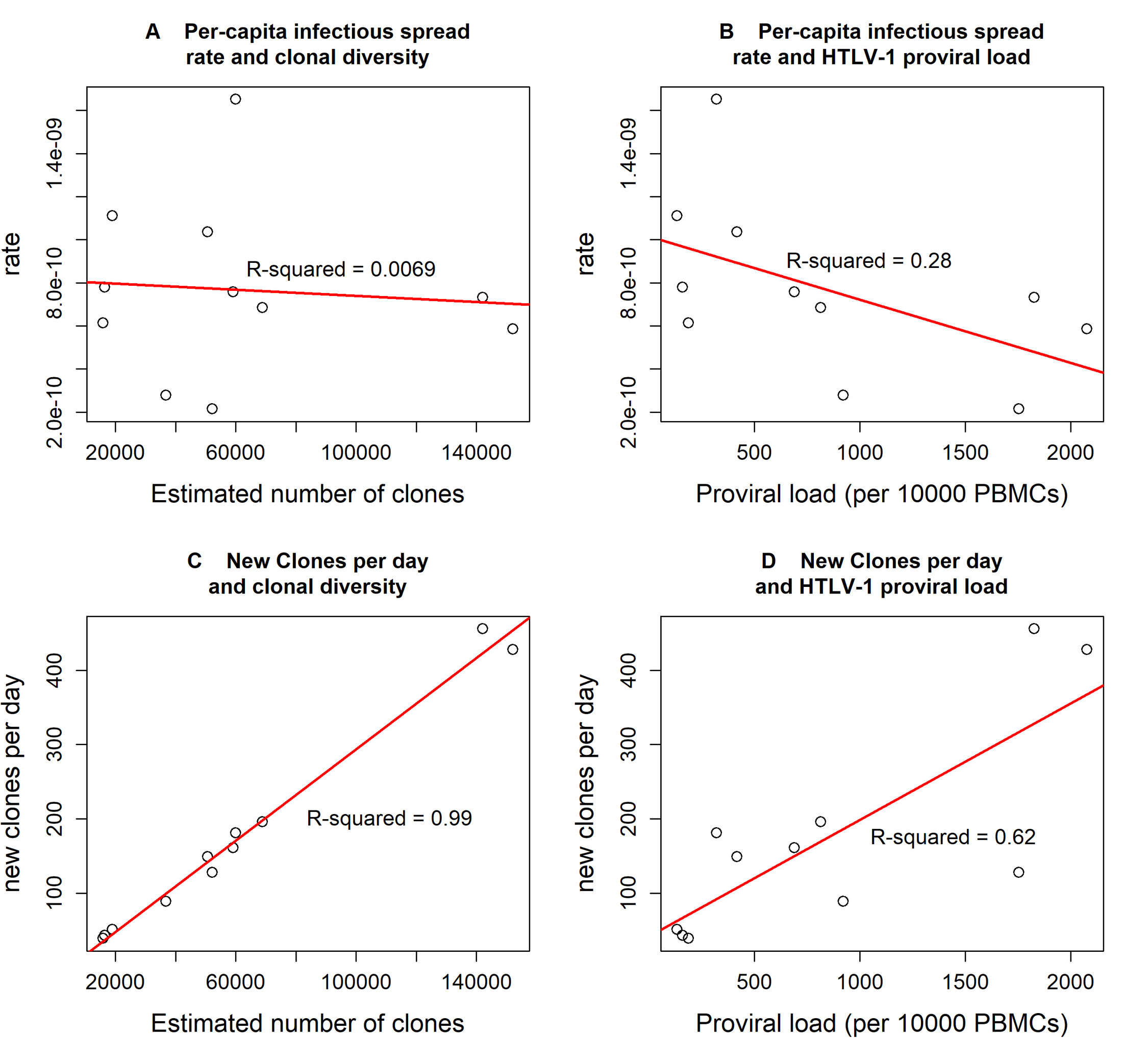

Supplement: S2 Fig — Infectious spread rate is not correlated with either the estimated diversity (A, R2 = 0.0069) during the chronic phase or proviral load (B, R2 = 0.28). However, unsurprisingly, the estimated number of new clones per day was strongly correlated with both the estimated diversity (C, R2 = 0.99) during the chronic phase or proviral load (D, R2 = 0.62) and proviral load. (TIF) [file pcbi.1007470.s008.tif]

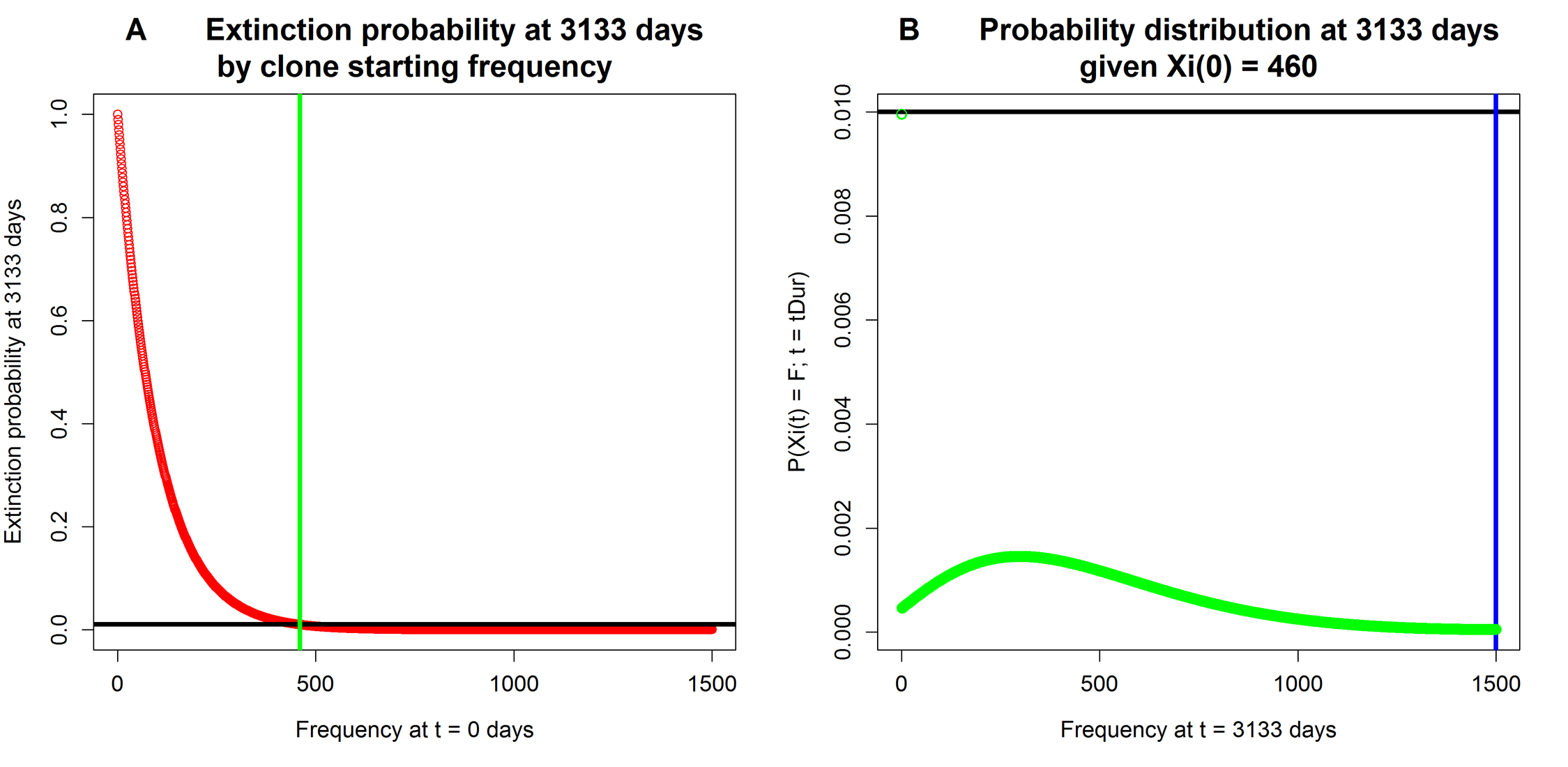

Supplement: S3 Fig — A Extinction probabilities by clone starting frequency (red) at tDur = 3133 days (given our values of infected cell proliferation and death parameters). Stochastic threshold frequency F = 460 (green) is chosen to be the minimum starting frequency such that a clone has less than a 1% (black line) chance of extinction. B Probability distribution of a clone at tDur = 3133 days given starting frequency F = 460. The upper limit τ (blue) is chosen so that this probability distribution is not significantly distorted. (TIF) [file pcbi.1007470.s009.tif]

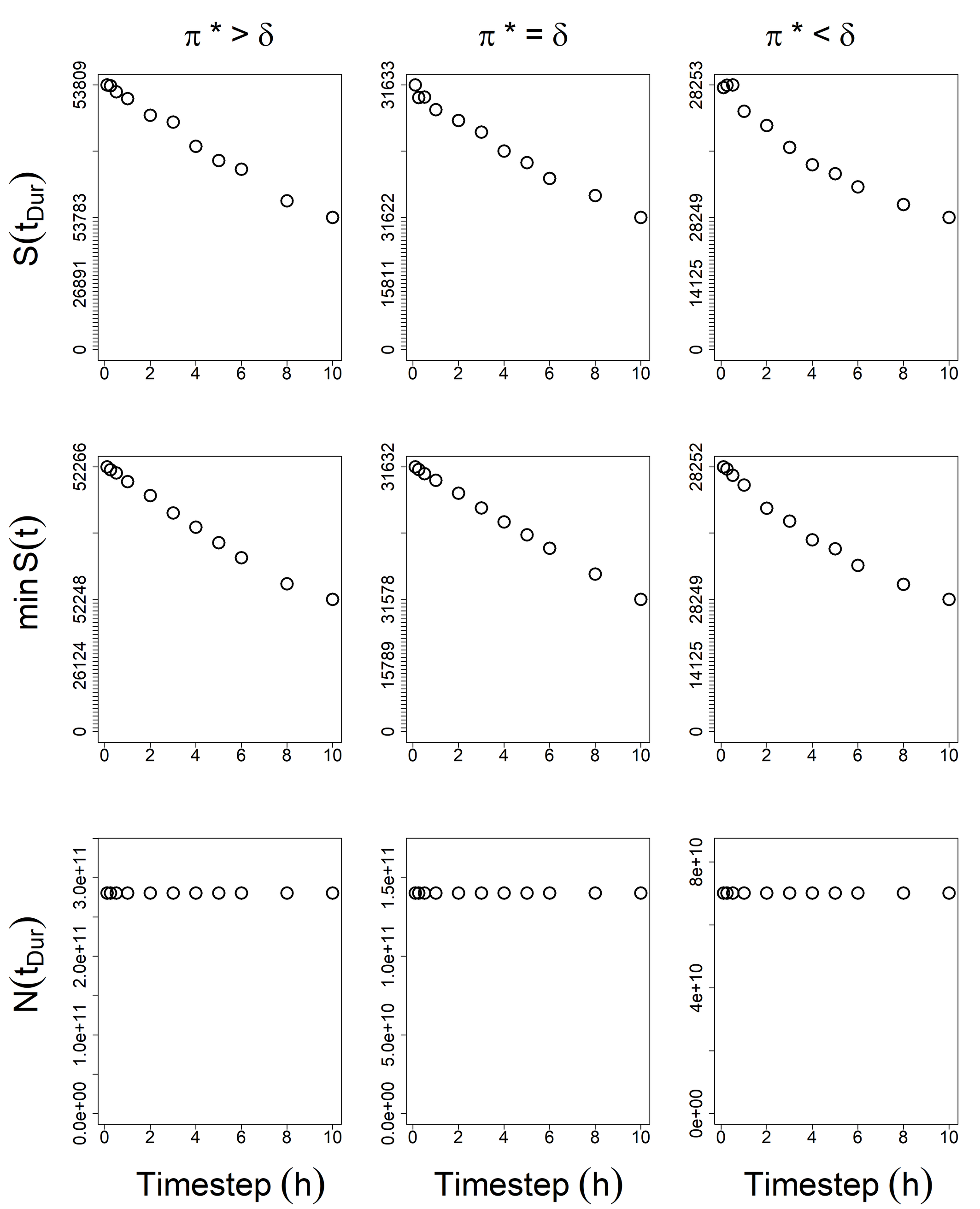

Supplement: S4 Fig — Expected diversity S(tDur) at given duration (tDur = 600 days) (first row); minimum diversity predicted throughout given duration (second row); and the number of infected cells N(tDur) predicted at tDur (third row), plotted against time step length h for three parameter sets: θG = {rI = 2 × 10^−11^, π* = 0.0316, δ = 0.5 * π*} (left column—“growth”); θE = {rI = 2 × 10^−11^, π* = 0.0316, δ = π*} (middle column—“equilibrium”); θD = {rI = 2 × 10^−11^, π* = 0.0316, δ = 2 * π*} (right column—“death”) for an example patient data set. Note that y-axes are split so that time step differences are visible. Time step effects are negligible for each observable. (TIF) [file pcbi.1007470.s010.tif]

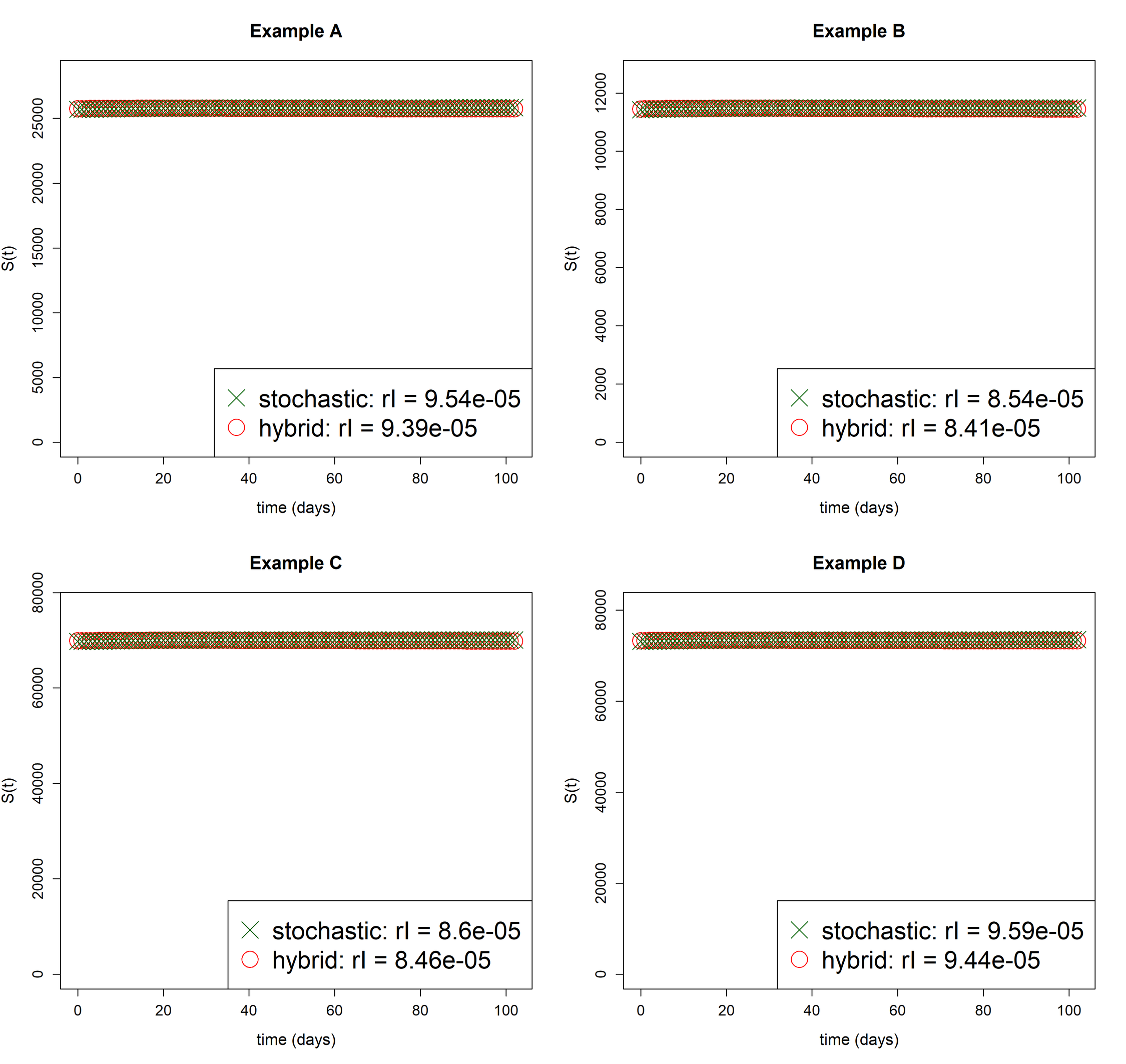

Supplement: S5 Fig — Reduced system omits all clones of size greater than 460 cells. For each patient, both the estimated number of clones over time and the fitted values of rI are very similar between the pure stochastic and the deterministic/stochastic hybrid model. (TIF) [file pcbi.1007470.s011.tif]

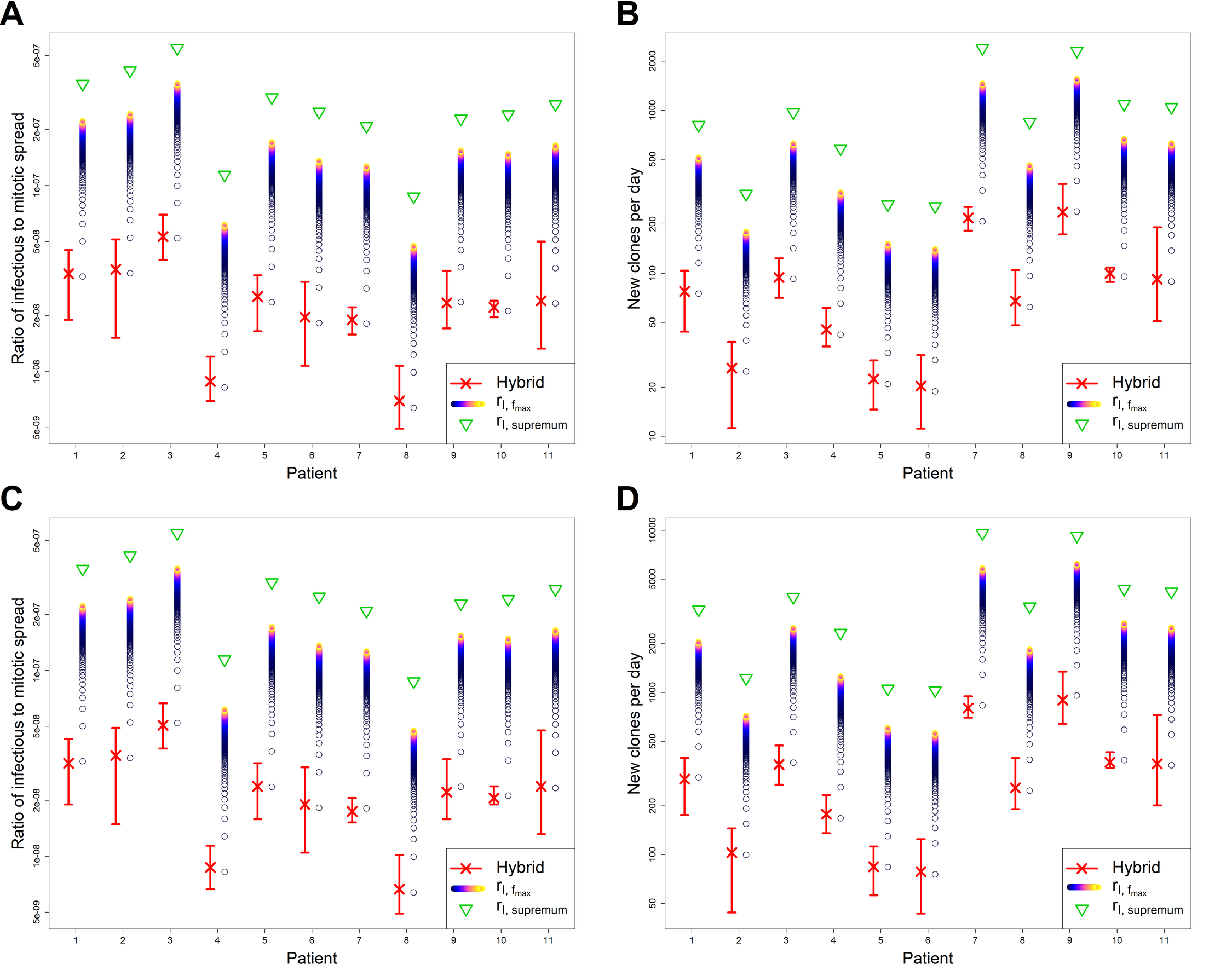

Supplement: S6 Fig — As per Fig 5. A, C Ratio of infectious spread to mitotic spread. B, D Number of new clones generated per day. A, B assume π* = δ = 0.0158, half the value in our main analysis. C, D assume π* = δ = 0.0632, twice the value in our main analysis. Estimates of the ratio of infectious spread to mitotic spread are almost identical. The infectious spread rate and therefore the number of new clones per day are sensitive to choice of proliferation and death parameters, although values are comparable. Upper bound approximations for fmax = 1 again match the more-detailed hybrid model. (TIF) [file pcbi.1007470.s012.tif]
